# Phenotypic Analysis, Molecular Characterization, and Antibiogram of Caries-Causing Bacteria Isolated from Dental Patients

**DOI:** 10.3390/microorganisms11081952

**Published:** 2023-07-31

**Authors:** Khushbu Farva, Huma Sattar, Hayat Ullah, Abdur Raziq, Muhammad Danish Mehmood, Afrasiab Khan Tareen, Imrana Niaz Sultan, Quratulaain Zohra, Muhammad Waseem Khan

**Affiliations:** 1 Institute of Molecular Biology and Biotechnology, The University of Lahore, Lahore 54000, Pakistan; 2 Metabolic Engineering Lab, Department of Biological Engineering, Utah State University, Logan, UT 84322, USA; 3 State Key Laboratory for Biology of Plant Diseases and Insect Pests, School of Bioengineering, Dalian University of Technology, Dalian 116024, China; 4 Department of Biotechnology, Balochistan University of Information Technology Engineering and Management Sciences, Quetta 87300, Pakistan; 5 Department of Biotechnology, Project of Sahara for Life Trust, The Sahara College Narowal, Punjab 51601, Pakistan

**Keywords:** antimicrobial activity, biofilm, dental caries, ethnomedicinal, herbal extracts, synthetic antimicrobial agents

## Abstract

Dental caries is a biofilm-mediated, sugar-driven, multifactorial, dynamic disease that results in the phasic demineralization and remineralization of dental hard tissues. Despite scientific advances in cariology, dental caries remains a severe global concern. The aim of this study was to determine the optimization of microbial and molecular techniques for the detection of cariogenic pathogens in dental caries patients, the prevalence of cariogenic bacteria on the basis of socioeconomic, climatological, and hygienic factors, and in vitro evaluation of the antimicrobial activity of selected synthetic antibiotics and herbal extracts. In this study, oral samples were collected from 900 patients for bacterial strain screening on a biochemical and molecular basis. Plant extracts, such as ginger, garlic, neem, tulsi, amla, and aloe vera, were used to check the antimicrobial activity against the isolated strains. Synthetic antimicrobial agents, such as penicillin, amoxicillin, erythromycin, clindamycin, metronidazole, doxycycline, ceftazidime, levofloxacin, and ciprofloxacin, were also used to access the antimicrobial activity. Among 900 patients, 63% were males and 37% were females, patients aged between 36 and 58 (45.7%) years were prone to disease, and the most common symptom was toothache (61%). For oral diseases, 21% used herbs, 36% used antibiotics, and 48% were self-medicated, owing to sweets consumption (60.66%) and fizzy drinks and fast food (51.56%). *Staphylococcus mutans* (29.11%) and *Streptococcus sobrinus* (28.11%) were found as the most abundant strains. Seven bacterial strains were successfully screened and predicted to be closely related to genera *S. sobrinus*, *S. mutans*, *Actinomyces naeslundii*, *Lactobacillus acidophilus*, *Eubacterium nodatum*, *Propionibacterium acidifaciens*, and *Treponema Pallidum*. Among plant extracts, the maximum zone of inhibition was recorded by ginger (22.36 mm) and amla (20.01 mm), while among synthetic antibiotics, ciprofloxacin and levofloxacin were most effective against all microbes. This study concluded that phyto extracts of ginger and amla were considered suitable alternatives to synthetic antibiotics to treat dental diseases.

## 1. Introduction

The oral cavity (the first part of the alimentary canal) is responsible for three primary functions: digestion, communication, and respiration. The structure of the oral cavity is critical in speech, perception of taste, and the first stages of digestion [1]. The oral cavity serves as the gateway to the digestive system. The masticatory apparatus is made up of teeth, which are fixed to the jaws [2]. In humans, the teeth are replaced once in contrast with non-mammalian vertebrates, where teeth are constantly replaced throughout life (polyphyodonty) [3]. A healthy tooth has three parts: crown, root, and neck. Pulp, dentine, enamel, and cementum are the structural parts of it [4]. Additionally, enamel is the major component of teeth. Enamel is the body’s toughest and most mineralized material made up of 96% mineral and 4% of water and protein [5]. Mouth bacteria in addition to several meals, snacks, soft drinks, and sweets may produce lactic acid. The acidic conditions progressively destroy the enamel, causing tooth cavities. This promotes bacterial penetration further into the tooth, aiding deterioration [6]. 

The mouth cavity is largely sterile at the time of birth. Within hours after birth, microbes are colonized in the mouth from the surrounding environment, and this colonization continues for months [6]. Some microorganisms, on the other hand, tend to remain in the cavity (a small number of people) for a short period of time. They are the members of the transitory flora [7]. Bacteria are the most common kind of microbes found in the oral cavity and are responsible for most infections. More than 30 bacterial genera have been identified in the human mouth [8], 25 of which are considered to be regular members of the oral flora. They are members of both aerobic and anaerobic, Gram-positive, and Gram-negative bacterial communities. Bacilli, cocci, treponemes, and mycoplasma are bacteria that may be found in the oral cavity [9]. Moreover, unhygienic oral conditions can result in plaque formation, composed of numerous bacteria comprising over 400 species, which tenaciously adhere to the tooth surface, resulting in the formation of a biofilm. This biofilm may turn on the inflammatory process, resulting in the ulceration of the gingival sulcular epithelium, hence bacteria get access to the bloodstream and cause infection [10]. Prolonged exposure to these germs might lead to tissue harm and, ultimately, systemic disorders, i.e., myocardial infarction [11,12].

Among one-third of all oral infections, aerobic microorganisms are found more frequently, although anaerobic ones are also present alongside them [13]. To better understand the process of disease and offer efficient antimicrobial therapy, it is essential to identify the bacteria involved precisely [14]. The biochemical and physical attributes of known and reference strains under optimal growth conditions are used to identify bacteria [15]. However, since phenotypic traits may alter under certain conditions, i.e., stress, even successful culturing does not guarantee accurate identification [16]. As a result of the advancement in technology, pathogen detection has increasingly included microscopy, immunological tests, and molecular approaches, in addition to traditional procedures. It was believed that molecular approaches, such as polymerase chain reaction (PCR)-based methods, would eventually eliminate the requirement for microscope examination, culturing, and immunological testing [17]. Molecular approaches, when compared to normal procedures, are simpler, quicker, and more sensitive than the later [18].

Antibiotics taken for a longer period of time may produce resistance even in low doses [19]. The bacteria *Staphylococcus aureus* is a good example of acquired resistance since it has developed resistance to a variety of medications, including vancomycin [20]. In addition, traditional medicines can also be used for oral infections. Traditional medicine is practiced by 80% of the people in Asia and Africa [20]. Herbal therapies are mostly used among the numerous types of traditional medicines available and are classified into many categories [21]. 

This study was designed for the optimization of microbial and molecular techniques for the detection of cariogenic pathogens in dental caries patients, the prevalence of cariogenic bacteria based on socioeconomic, climatological, and hygienic factors, and to evaluate the antimicrobial activity of selected synthetic antibiotics and herbal extracts.

## 2. Materials and Methods

### 2.1. Sample Selection Criteria

Samples from volunteers who visited the operative departments of the University College of Medicine and Dentistry (UCMD) for their dental checkups were included in this study. A total of 900 patients were selected from the volunteers who met the inclusion criteria. The volunteers were accessed through a questionnaire that includes dental clinical and socioeconomical values, such as age, gender, clinical history, and other inquiries related to teeth health. Patients’ complaints of dental sensitivity, pain in teeth, calculus, and bad breath were included in the study.

### 2.2. Collection of Clinical Material

The clinical material was collected in two phases. In the first phase, early morning saliva samples of patients were collected in sterile tubes after 5 min chewing and continuous salivation produced by 1 cm^3^ paraffin wax [22]. In the second phase, a pooled plaque specimen was collected onto cotton swabs. Sterile swabs were rubbed across the facial and lingual surfaces of maxillary and mandibular anterior teeth and the other across the occlusal surfaces of maxillary and mandibular molars. Swab samples were placed in a screw-capped sterile vial containing normal saline. Carious scraping was obtained from the carious tooth [23].

### 2.3. Determining Dental Caries Susceptibility

Snyder’s test was used to measure the susceptibility to dental caries [24]. The saliva sample (0.2 mL) was inoculated into Snyder agar, vortexed, and incubated at 37 °C for 24–72 h. Susceptibility to dental caries was determined by the color change from green to yellow that occurred during 24 h, 48 h, and 72 h, respectively.

### 2.4. Selection of Bacterial Strains

The intricate ecology of microorganisms in the human mouth is made up of hundreds of bacterial species. There is a lot of information out there on the role of particular species in pathogenesis; however, the system-level processes that lead to illness are still a mystery [25]. Based on studies, from the complex oral microbial flora some bacterial stains (*Streptococcus sobrinus*, *Staphylococcus mutans*, *Actinomyces naeslundii*, *Lactobacillus acidophilus*, *Eubacterium nodatum*, *Propionibacterium acidifaciens,* and *Treponema pallidum*) were selected due to least study present in the selected area of study.

### 2.5. Bacterial Isolation and Identification

Plaque sample (0.1 mL) was streaked on sterile 5% blood agar and Mitis Salivarius Bacitracin (MSB) Agar, incubated at 37 °C for 24 h and 48 h. Pure cultures were produced by sub-culturing a single colony on fresh blood agar and MSB agar and cultivating the resultant cultures. A pure colony was suspended in saline and a loopful of this suspension was streaked on blood agar, incubated at 37 °C for 24 h [26]. Selective colonies were picked for Gram staining after observing colony morphology and were evaluated for the biochemical reaction using a commercially available rapid bacterial detection kit (API-Biomeurix France) [27,28].

### 2.6. Biochemical Test for Bacterial Strains

The bacterial strains were tested biochemically through fermentation tests [29], enzyme activity tests [30], and physiological tests [31]. In the fermentation process, different compounds, such as glucose, lactose, galactose, mannose, maltose, sucrose, sorbitol, inulin, raffinose, araninose, trehalose, xylose, arabinose, rhamnose, raffinose, trehalose, were tested. Enzyme activity tests were based on the hydrolysis of esculin, arginine, urea, starch, and ONPG (O-nitrophenyl-beta-D-galactopyranoside) separately, and other tests, including Voges Proskauer test, indole production, methyl red test, citrate utilization, nitrate reduction, H_2_S production test, casein hydrolysis, gelatin hydrolysis, phenylalanine deamination, nitrate reduction, lipase production, lysine decarboxylation, and ornithine decarboxylation, were analyzed. Physiological tests were performed with bacterial growth in 2%, 5%, 7%, and 10% NaCl.

### 2.7. Nucleic Acid Extraction and Estimation and PCR Amplification

Bacterial nucleic acid was extracted and estimated using the standard protocols of Wright et al. (2017) [32] and Wilson (2001) [33] and User Manual Nano-Drop 2000, respectively. Specific primers were designed for each isolate based on the previously published literature (Table S1). The bands of specific lengths were visualized by gel electrophoresis.

PCR (50 µL) contained 10 ng of DNA, 1 µL of PCR buffer,1.5 U of Taq polymerase, 0.2 µL of each primer (Table S1), 200 lM of each dNTPs, and 1.75 mM of MgCl_2_. The DNA amplification conditions were initial denaturation at 95 °C for 5 min, 35 cycles of 95 °C for 1 min, 55 °C for 40 s, 72 °C for 1 min 40 s, and final extension at 72 °C for 10 min. The PCR products were analyzed on 1% agarose gel electrophoresis.

### 2.8. Selection of Herbal Plants Having Antimicrobial Activity

Seven herbal plants, such as Garlic cloves (*Allium sativum*) [34], Ginger-dried rhizome (*Zingiber officinale*) [35,36], Neem seeds (*Azadirachta indica*) [37,38], Tulsi leaves (*Ocimum tenuiflorum*) [39], Amla bark (*Phyllanthus emblica*) [40], and Aloe vera leaf (*Aloe vera*) [41,42], were selected for antimicrobial activity against isolated bacterial strains. Plants extracts (aqueous, methanolic, and ethanolic) were prepared according to the protocols mentioned in Table 1. The physical features of the medicinal plant extracts were checked and measured in grams.

### 2.9. Phytochemical Analysis of Extracts

A preliminary phytochemical study was carried out to determine the active chemical principles of the selected plant under investigation. For this purpose, various tests, such as alkaloids, carbohydrates, amino acids, saponins, tannins, flavonoids, anthraquinones, cardiac glycosides, fats, and fixed oils detection tests, were performed according to [61,62,63,64] protocols.

### 2.10. Antibacterial Activity Study of Herbal Extracts and Synthetic Agents

#### 2.10.1. Preparation of Media for Bacterial Growths

Different types of growth media with modification were used for bacterial growth as discussed in Table 2.

#### 2.10.2. Determination of Zone of Inhibition by Herbal Extracts and Synthetic Agents

Agar well diffusion technique [72] was used to measure the antibacterial activity of the plant extract tested against each isolated bacterial strain. The freshly isolated colony of bacteria was suspended in sterile saline to achieve a turbidity of 0.5 McFarland standard after being isolated overnight, and 0.1 mL of this suspension was applied to the Muller Hinton agar. Each medicinal extract (aqueous, methanolic, and ethanolic) was added to wells (8 mm in diameter) at concentrations of 25 µg/mL, 50 µg/mL, 75 µg/mL, and 100 µg/mL and incubated at 37 °C for 24 h. The zone of inhibition was determined in millimeters (mm). Triplicates of each extract were taken. Selected antibiotics, such as Penicillin and Amoxicillin 25 µg/mL each; Erythromycin and Clindamycin 5 µg/mL each; Metronidazole, Ceftazidime, Levofloxacin, and Ciprofloxacin 30 µg/mL each; and Doxycycline 5 µg/mL, were also used with the plant extracts to measure the antibacterial activity.

### 2.11. Statistical Analysis

Graph Pad Prism 8, Statistica version 8.1, and Microsoft Excel software (2018) were used to analyze the data statistically. Results were displayed in mean ± standard deviation of triplicate values for each test. The confidence level was set at 95% for the three-way ANOVA and values were considered significant when *p* ˂ 0.05.

## 3. Results

### 3.1. Socioeconomic and Clinical Values of Patients

Among 900 patients, 63% (N = 566) were males and 37% (N = 334) were females. The age of study participants ranged from 18 to 70 years, with the majority of patients, i.e., 45.7% (412 patients), falling into the middle-age category (36–58 years). Based on patients’ initial symptoms, 61% (N = 545) had just pain in their teeth, 12% (N = 109) reported bleeding in their gums, and 27% (N = 246) were having both problems, while 57.2% (N = 515) were having teeth sensitivity. Tooth cleaning was reported by 70% (N = 630) of the participants and 76% (N = 683) wash their mouth (40% weekly, 28% monthly, and 32% randomly).

#### 3.1.1. Herbal and Antibiotics Usage

**Figure 1:**
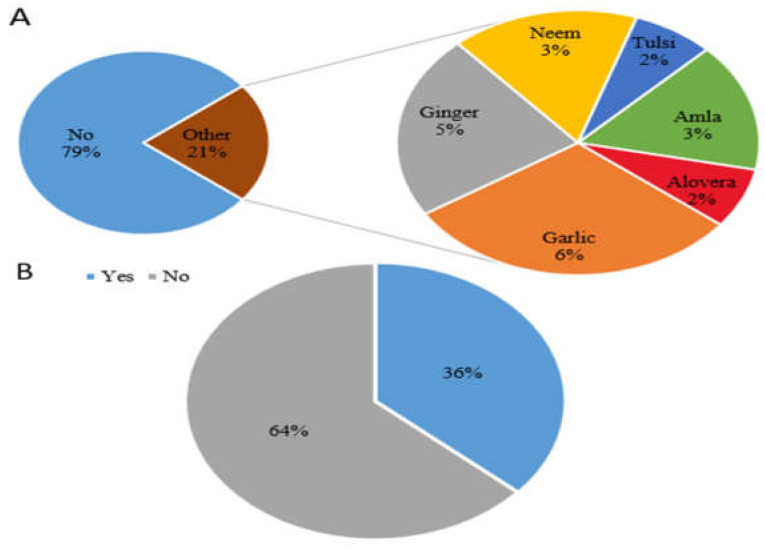
(**A**) Herbs used for oral treatment and (**B**) participants using antibiotics.

Among patients, 21% (N = 189) used herbs for dental problems. Among the herbs, garlic (6%) was the most commonly used, followed by ginger (5%), neem (3%), amla (3%) tulsi (2%), and aloe vera (2%) (Figure 1A). Regarding antibiotics, 36% (N = 328) of participants used antibiotics for the treatment of oral diseases, while 64% (N = 572) never used antibiotics for oral diseases (Figure 1B).

#### 3.1.2. Usage of Medicine Other Than Prescription

**Figure 2:**
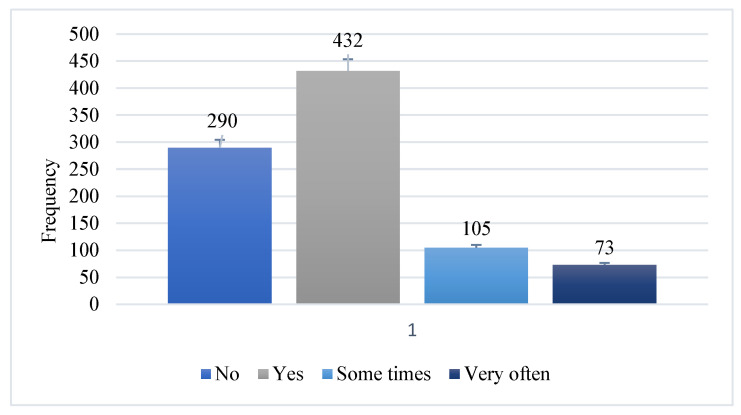
Participant’s attitude towards self-medication.

Among all study participants, 32.22% (N = 290) did not ever self-medicate to treat dental problems, while a large number (48%, N = 432) self-medicated when they had any oral infection. Additionally, 11.67% (N = 105) sometimes self-medicated, while only 8.11% (N = 73) rarely used medicine as a treatment for dental problems on their own (Figure 2).

#### 3.1.3. Dental Visits by the Patients

**Figure 3:**
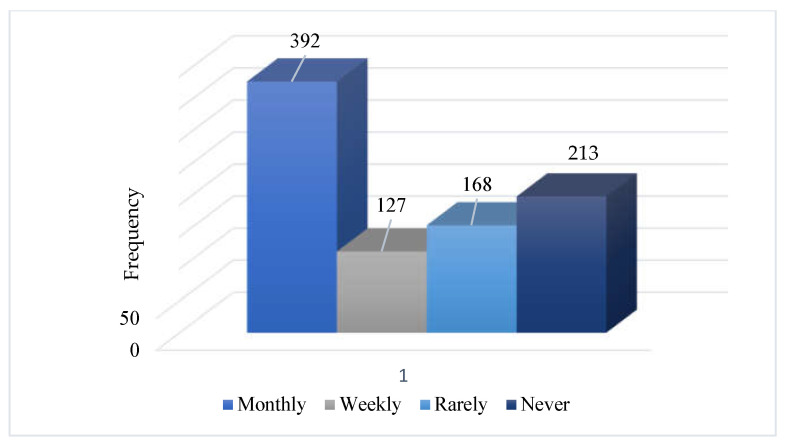
Visit attitude of participants towards the clinic.

Among all study participants, 43.56% (N = 392) preferred to visit the clinic (14.11% monthly, 18.67% weekly, and 18.67% rarely), while 23.67% (N = 213) never visited dentists (Figure 3).

#### 3.1.4. History and Screening Tests for Hepatitis C

**Figure 4:**
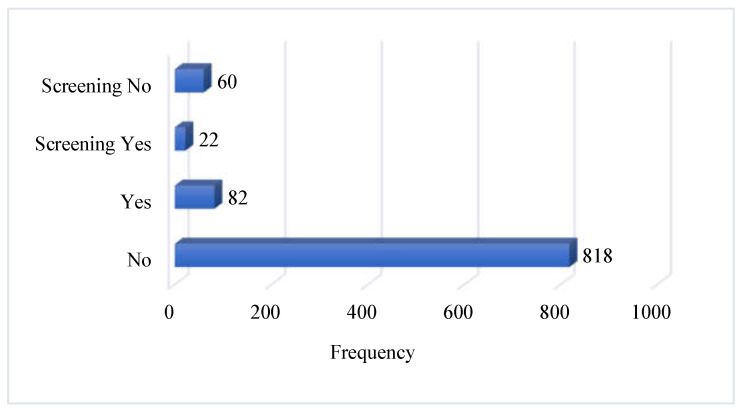
History and screening of HCV by the participants.

Among the studied participants, 90.89% (N = 818) did not have any history of hepatitis C, while 9.11% (N = 82) had a history of hepatitis C. Among the patients with hepatitis C, 26.83% (N = 22) had regular screening, while 73.17% (N = 60) did not have screening tests on a regular basis (Figure 4).

#### 3.1.5. Sweet and Fast Foods Intake

**Figure 5:**
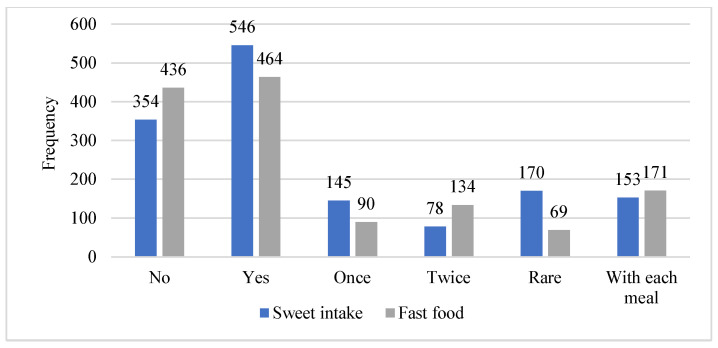
Sweet and fast foods intake by participants.

Among the study participants, 60.66% (N = 546) consume sweets; among them, 26.55% (N = 145) consume once a day, 14.28% (N = 78) twice a day, 31.13% (170) very rarely, and 28% (N = 153) with each meal, whereas 39.33% (N = 354) were not consuming any sweets. Regarding fast foods, 48.44% (N = 436) were not consuming fast food and drinks; however, 51.56% (N = 464) consumed fizzy drinks and fast foods, among them 36.85% (N = 171) had fast food and drinks once a day, 28.87% (N = 134) had twice a day, 19.40% (N = 90) drinks once a day, and 14.87% (N = 69) had fast food and drinks very rarely (Figure 5).

#### 3.1.6. Type of Bacteria Identified

**Figure 6:**
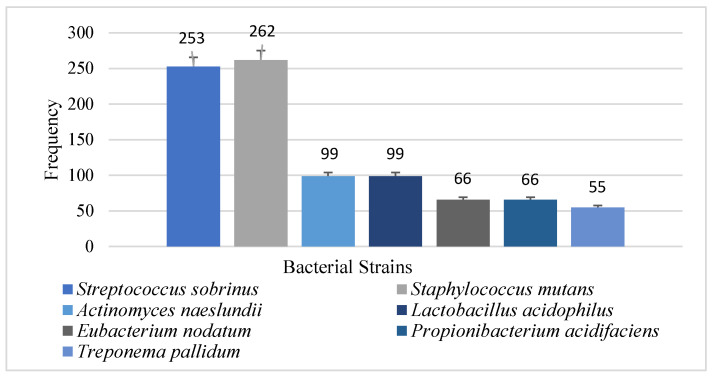
Isolated bacteria from the participants.

Among the study participants, the most abundant strains were *S. mutans* and *S. sobrinus*, accounting for 29.11% (N = 262) and 28.11% (N = 253), respectively; however, *A. naeslundii* and *L. acidophilus* were 11% (N = 99) each, *E. nodatum* and *P. acidifaciens* were 7.3% (N = 66) each, and *T. pallidum* was 6.1% (N = 55) (Figure 6).

#### 3.1.7. Comparison between Herbal Treatment and Suggested Dentist Treatment Plan

**Figure 7:**
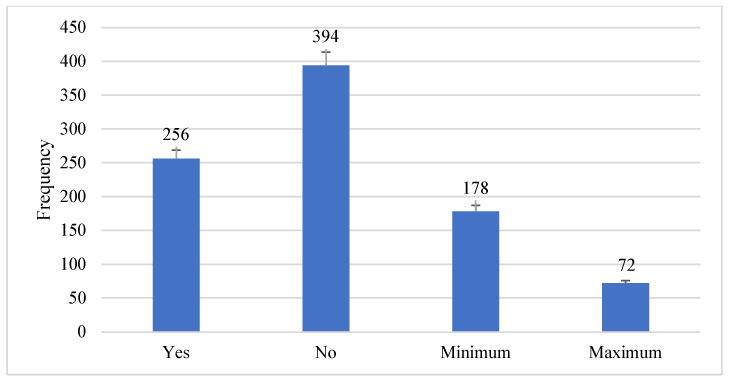
Comparison between herbal and prescribed medicines.

Herbal medicine as a traditional and local treatment is used for various diseases. The attitudes towards the best treatment for dental caries were tried to be determined by the participants. Figure 7 shows the detailed attitude of participants while comparing herbal medicine with prescribed medicine.

### 3.2. Selection of Bacterial Strains

Based on the microscopic and macroscopic analysis, such as colony morphology, Gram staining, biochemical, and physiological tests, seven bacterial strains, DKF 001, DKF 002, DKF 003, DKF 004, DKF 005, DKF 006, and DKF 007, were isolated and predicted to be closely related to the genera *S. sobrinus*, *S. mutans*, *A. naeslundii*, *L. acidophilus*, *E. nodatum*, *P. acidifaciens*, and *T. Pallidum*, respectively. Furthermore, gene sequencing of the strains is required to confirm the genera and exactly find out the species belonging to these genera.

### 3.3. Isolation of Bacterial Strains, Gram Staining, and Morphological Characteristics

**Figure 8:**
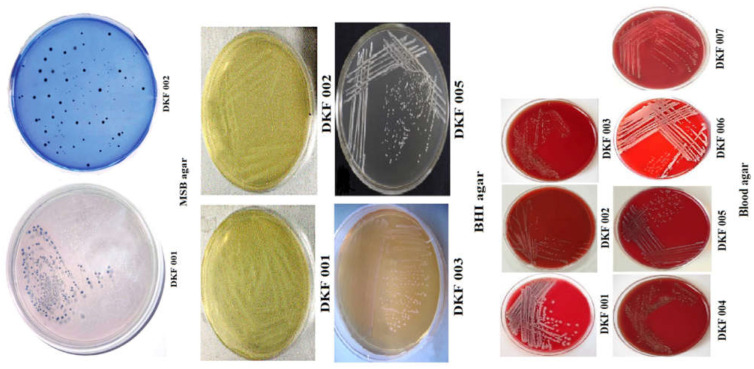
The growth of bacterial strains on MSB agar, BHI agar, and Blood agar media.

The growth pattern of bacterial isolates on MSB, blood agar, BHI agar, and SB-20M agar was observed for each strain (Figure 8; Table 3). The Gram-staining pattern, including color, shape, and size, was observed for each strain. 

### 3.4. Identification of Bacterial Strains

#### 3.4.1. Biochemical Identification

##### Enzyme Activity Analysis

DKF 003 showed highly positive results for esculin, urease, starch, and OPNG hydrolysis analysis. It was able to reduce nitrate, and hydrogen sulfide gas was produced and positive for phenylalanine dehydrogenase. The enzymatic activity of other isolates is discussed in Table 4.

##### Sugar Fermentation Analysis of Isolated Strains

DKF 001 was positive for glucose, lactose, mannose, sucrose, raffinose, and maltose. DKF 002 fermented all tested sugars, except xylose, arabinose, rhamnose, and dulcitol. DKF 003 fermented glucose, lactose, mannose, sucrose, sorbitol, inulin, raffinose, maltose, and trehalose. DKF 004 fermented all tested sugars, except sorbitol, inulin, trehalose, and dulcitol. DKF 005 fermented glucose, lactose, sucrose, and maltose. DKF 006 fermented glucose, lactose, mannose, sucrose, raffinose, maltose, arabinose, and fructose. DKF 007 fermented lactose, sucrose, sorbitol, maltose, trehalose, xylose, and dulcitol (Table 5).

#### 3.4.2. Physiological Analysis of Isolated Strains

All isolated strains showed positive growth under 2% and 5% NaCl concentrations (except DKF 001 and DKF 007 for 5%), while DKF 005 exhibited positive growth at all concentrations (Table 6).

The catalase-negative test indicated that DKF 001 and DKF 002 were streptococci and were able to grow on Mitiis-Salivarius (MS) agar. These strains’ growth on SB-20M media differentiated them as *S. mutans* and *S. sobrinus,* as reported by Saravia et al. [73].

#### 3.4.3. Molecular Identification

**Figure 9:**
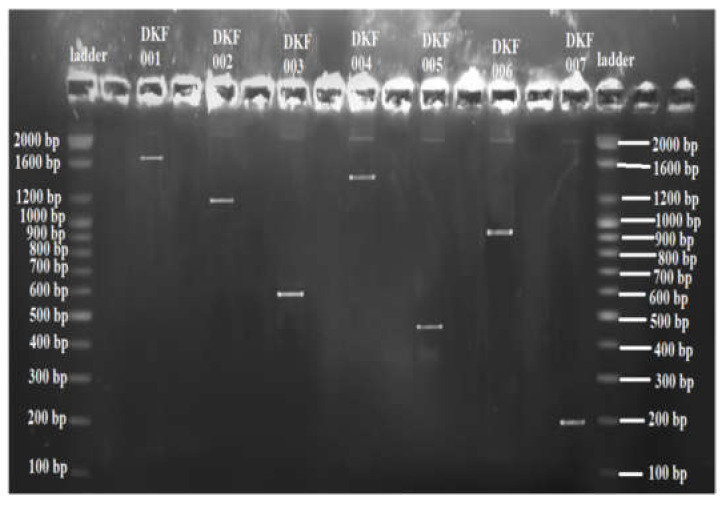
Gel-electrophoresis analysis of PCR products of isolated bacterial strains.

Gel-electrophoresis analysis indicated that 16S RNA gene amplification sizes of DKF 001, DKF 002, DKF 003, DKF 004, DKF 005, DKF 006, DKF 007 were 1.61 kbp, 1.272 kbp, 0.6 kbp, 1.5 kbp, 0.492 kbp, 0.950 kbp and 0.209 kbp, respectively, as shown in Figure 9.

### 3.5. Physiochemical Properties of Selected Herbal Plants and Their Extracts

#### 3.5.1. Physical Properties of Extracts

The results of plant extracts’ physical features, such as color, odor, and consistency, are given in Table 7.

#### 3.5.2. Percentage Yield of Medicinal Plant Extracts

The percentage of plant extracts in aqueous, methanol, and ethanol solvents was described in Table 8. The maximum yield of garlic was seen in methanol (34%). In ethanol, the yield was found to be 32.93% and in water, it has only a 26.07% yield. Methanolic extract (62.93%) > ethanolic extract (43.6%) > aqueous extract (56.23%) were the yields of ginger. In Neem, the trend of yield was ethanolic extract (40.87%) > methanolic extract (35.47%) > aqueous extract (32.47%). Tulsi, amla, and aloe vera showed maximum yield in aqueous media (47.33%, 43.14%, and 51.27%, respectively).

### 3.6. Phytochemical Analysis of Extracts

The preliminary phytochemical screening revealed the presence of alkaloids and saponins in all plant extracts. Carbohydrates, tannins, flavonoids, cardiac glycosides, fats and fixed oils, proteins, and amino acids were also found in different extracts of the desired plants; details of phytochemicals have been given in Table 9.

#### 3.6.1. Alkaloids Detection

Methanolic and ethanolic extracts of garlic have alkaloids, while in ginger, only ethanolic extract showed the presence of alkaloids. Both aqueous and methanolic extracts of neem showed the presence of alkaloids. Alkaloids were found in the ethanolic extract of tulsi and in the aqueous extracts of aloe vera. 

#### 3.6.2. Detection of Carbohydrates, Proteins, and Amino Acids

All the extracts of herbal plants, except the aqueous extract of neem, aqueous and methanolic extracts of tulsi, methanolic and ethanolic extracts of amla, and methanolic extract of aloe vera, showed the presence of carbohydrates. Only the aqueous extract of garlic and amla, ethanolic extract of ginger, amla, and aloe vera, and methanolic and ethanolic extracts of neem and tulsi showed the presence of proteins. The aqueous extracts of garlic, ginger, neem, and amla, methanolic extract of neem, tulsi, and aloe vera, and ethanolic extract of tulsi showed the presence of amino acids.

#### 3.6.3. Detection of Saponins, Tannins, and Flavonoids

The aqueous extract of ginger, methanolic extract of neem, and ethanolic extracts of tulsi, amla, and aloe vera showed negative results during the saponins detection in the extracts of herbal plants. Tannins were also not present in the aqueous extract of garlic, ginger, and neem, methanolic extracts of amla and tulsi, and ethanolic extracts of garlic and aloe vera. Flavonoids were present in most of the extracts, except the methanolic extract of garlic, neem, tulsi, and amla; among the aqueous extracts, flavonoids were absent in ginger, amla, and aloe vera. All ethanolic extracts had tannins.

#### 3.6.4. Detection of Cardiac Glycosides, Fats, and Fixed Oils

All the garlic extracts were positively detected for cardiac glycosides, while only the methanolic extract of ginger had cardiac glycosides. In neem and tulsi, except the aqueous extract, the others have cardiac glycosides. In amla, only ethanolic extract showed positivity to the cardiac glycosides. Aqueous and ethanolic extracts of aloe vera also showed a positive response during testing. Fats and fixed oils were also not found in the methanolic extract of garlic, aqueous extract of ginger, aqueous and methanolic extracts of neem, and aqueous extracts of amla and aloe vera.

### 3.7. Antibacterial Activity by Herbal Plants

The maximum zone of inhibition was recorded against *S. sobrinus*, *S. mutans*, *A. naeslundii*, *L. acidophilus*, *E. nodatum*, *P. acidifaciens*, and *T. pallidum* for all plant extracts. Against *S. sobrinus*, the maximum activity from aqueous extracts (20.01 mm) was recorded by amla (100 mg/mL); methanolic extract (20.94 mm) by ginger (100 mg/mL); and ethanolic extracts by ginger (100 mg/mL). Against *S. mutans*, the maximum activity from aqueous extracts (18.01 mm) was recorded by amla (100 mg/mL); methanolic extract (16.86 mm) by neem (100 mg/mL); and ethanolic extracts (21.95 mm) by ginger (100 mg/mL). Against *A. naeslundii*, the maximum activity from aqueous extracts (17.09 mm) was recorded by ginger (75 mg/mL); methanolic extract (16.93 mm) by tulsi (100 mg/mL); and ethanolic extracts (15.79 mm) by aloe vera (100 mg/mL). Against *L. acidophilus*, the maximum activity from aqueous extracts (17.49 mm) was recorded by ginger (100 mg/mL); methanolic extract (16.93 mm) by neem (100 mg/mL); and ethanolic extracts (15.79 mm) by garlic (100 mg/mL). Against *E. nodatum*, the maximum activity from aqueous extracts (17.18 mm) was recorded by garlic (75 mg/mL); methanolic extract (17 mm) by ginger (100 mg/mL); and ethanolic extracts (15.87 mm) by neem (100 mg/mL). Against *P. acidifaciens*, the maximum activity from methanolic extract (16.93 mm) was recorded by ginger (100 mg/mL) and ethanolic extracts (15.81 mm) by garlic (100 mg/mL). Against *T. pallidum*, the maximum activity from aqueous extracts (14.89 mm) was recorded by garlic (100 mg/mL); methanolic extract (15.87 mm) by tulsi (100 mg/mL); and ethanolic extracts (17.53 mm) by garlic (100 mg/mL) (Table 10). 

### 3.8. Antibacterial Activity by Synthetic Antibiotics

Ciprofloxacin and levofloxacin were most effective against all microbes. Erythromycin had least activity against *S. sobrinus* (14.31 mm) and *S. mutans* (15.03 mm). Against *A. naeslundii,* ciprofloxacin showed the maximum inhibition (29.8 mm), while amoxicillin (25 mg/mL) showed the minimum inhibition (9.1 mm). Against *L. acidophilus* and *E. nodatum*, amoxicillin exhibited inhibition activity of 12.3 mm and 15.4 mm, respectively. Penicillin was found to be least effective against *P. acidifaciens* (7.3 mm). Against *T. pallidum*, penicillin and amoxicillin indicated the lowest activity (7.8 mm) (Table 11)**.** The order of antimicrobial activity against the oral microbes included in this study was *S. sobrinus* > *S. mutans* > *E. nodatum* > *L. acidophilus* > *A. naeslundii* > *T. pallidum* > *P. acidifaciens*. For amoxicillin, *S. mutans* > *S. sobrinus* > *E. nodatum* > *L. acidophilus* > *A. naeslundii* > *P. acidifaciens* > *T. pallidum*.

**Erythromycin:** E. nodatum > S. mutans > L. acidophilus > S. sobrinus > A. naeslundii > P. acidifaciens > T. pallidum.

**Clindamycin:** S. sobrinus > S. mutans > E. nodatum > L. acidophilus > A. naeslundii > T. pallidum > P. acidifaciens.

**Metronidazole:** L. acidophilus > S. mutans > A. naeslundii > E. nodatum > S. sobrinus > T. pallidum > P. acidifaciens

**Doxycycline:** E. nodatum > S. mutans > L. acidophilus > S. sobrinus > A. naeslundii > P. acidifaciens > T. pallidum.

**Ceftazidime:** S. mutans > S. sobrinus > A. naeslundii > L. acidophilus > E. nodatum > P. acidifaciens > T. pallidum.

**Levofloxacin:** S. sobrinus > E. nodatum > S. mutans > A. naeslundii > L. acidophilus > P. acidifaciens > T. pallidum.

**Ciprofloxacin:** S. sobrinus > A. naeslundii > E. nodatum > S. mutans > L. acidophilus > P. acidifaciens > T. pallidum.

### 3.9. Statistical Analysis

ANOVA was conducted for the antibacterial activity of herbal extracts against *S. sobrinus* (Table S2), *S. mutans* (Table S3), *A. naeslundii* (Table S4), *L. acidophilus* (Table S5), *E. nodatum* (Table S6), *P. acidifaciens* (Table S7), and *T. pallidum* (Table S8). Tables S2–S8 indicate that the values are statistically significant when comparing the strength of extract × plant, type of extracts × strength of extract, and type of extracts × plant. All results are statistically significant with *p* ˂ 0.05.

## 4. Discussion

Dental caries and associated disorders are the most frequent diseases to be found in people worldwide [74]. The incidence of these illnesses is growing as a result of the shift in eating habits among individuals. Dental caries is a multifactorial illness that affects both the teeth and the gums [75]. The condition is impacted by a variety of variables, such as age, gender, food, microbiota in the mouth, salivary flow, tooth shape, and genetic tendency. In India, the prevalence of dental caries is estimated to be between 60% and 84% [76]; in Pakistan, the prevalence is estimated to be 56% [77]. 

Several experiments on oral microorganisms and their products have gained acceptance in the prediction of dental caries. These studies focused on Lactobacilli count and *S. mutans*, which are linked to dental caries. Although dietary carbohydrates are important etiological and predisposing variables for dental caries [78], the link between Lactobacilli count and the Snyder test positivity was determined by Snyder and Clarke [79]. They claimed that when Lactobacilli count exceeded 10,000 L/cc in saliva, 33.4% of samples tested positive in 24 h and 90% in 48 h.

A study conducted in Karachi, Pakistan reported that the majority of children between the ages of 9 and 18 were suffering from caries, with >40% of those suffering from the condition going untreated. The results of this study are not in line with our study; more individuals in the middle-aged group were affected as per our study results [80]. 

According to our study results, males are found to have more prevalence of caries as compared to females. The high prevalence in males may be due to different habitual patterns like smoking, more intake of sweets, and others [81,82]. A significant relationship was found between oral hygiene and dental caries; to maintain oral hygiene, it is necessary to adopt daily brushing and avoid sugary foods (refined) [83].

Dental caries is a serious public health concern in many areas of the globe, and the mechanical removal of oral biofilms is still the first line of defense against the development of caries and periodontal disease. Antibiotics are used to combat caries, but due to antibiotic resistance, the administration of antibiotics will not be sufficient to entirely block demineralization [84].

Bacterial antibiotics are frequently utilized in the treatment of dental caries and other dental-related disorders, both therapeutically and prophylactically [85]. Dental surgeons commonly prescribe antibiotics because they are concerned that the oral cavity, which ordinarily contains a large number of microorganisms as part of the natural flora and which might cause infections in their patients, would get infected [86].

Diet has an important impact on the development of dental caries and the degradation of enamel. Dental caries is a complex illness that emerges from interactions among a susceptible host, caries-related microorganisms, and cariogenic foods [87]. Organic acids that develop in the dental plaque are responsible for the demineralization of the tooth enamel and dentine, which is caused by anaerobic microbes metabolizing carbohydrates from the diet [88].

Acidogenic and cariogenic properties are present in soft drinks due to the presence of both acids and sugars, which may result in tooth caries and enamel loss [89]. According to the findings of many research studies, consuming soft drinks is associated with an increased risk of dental caries and erosion. Children aged 2–10 years who drank a high volume of carbonated soft drinks also had a considerably greater prevalence of dental caries than children who had a high volume of juice, milk, and water in their diet [90].

The biochemical analysis including sugar fermentation and enzyme activity was in agreement with the data reported by Soumya and Nampoothiri [91]. DKF 007 after the Gram staining was predicted to be the species belonging to *Treponema*. However, the spirochetes are difficult to culture on media and repeated culturing is required to obtain colonies. This strain was unable to grow on media for biochemical analysis. The difficulty in dealing with this organism in the microbiology lab has already been reported [92]. The findings were consistent with earlier research. Since different commercial and in-house systems have varied substrate specificities, buffering capacities, and therefore sensitivities, it might be challenging to interpret previously published results on enzyme reactions and fermentation studies. It is critical to understand the roles that these Gram-positive rods play in oral and non-oral environments and diseases, even if they are difficult to identify down to the species level.

According to the literature, *S. mutans* is more common in the oral cavity than *S. sobrinus* [93]. *S. mutans* has been implicated as one of the most important etiological agents for dental caries. Literature studies have found a positive relationship between caries experience and the presence or degree of *S. mutans* in saliva or plaque [94]. The incidence of caries is higher when *S. mutans* and *S. sobrinus* are present in the same oral microbiota. Okada et al. demonstrated that preschool children who have both *S. mutans* and *S. sobrinus* in their plaque samples have a higher incidence of dental caries than those who only have *S. mutans* [95]. It has also been demonstrated that *S. sobrinus*, in contrast to *S. mutans*, has not been discovered in any healthy control patients, and it may be a more effective caries-causing agent than *S. mutans*. Based on the current data, it is possible that the true incidence of *S. sobrinus* is higher than what has been believed [96]. 

The presence of significant levels of ureolytic activity in human dental plaque has been linked to the maintenance of plaque pH homeostasis and plaque ecology and the development of dental caries, calculus, and periodontal disease. Although the organisms responsible for this activity have not been identified with certainty, the molecular aspects of ureolysis in dental plaque have not been studied in depth [97]. While several dental plaque organisms have demonstrated ureolytic activity when isolated, *Actinomyces* strains are the most abundant. They can be found in high concentrations in both supragingival and subgingival plaque, indicating that they have the potential to be significant contributors to total plaque ureolysis. Thus, specific primers for urease gene *ureC* were designed to amplify the DNA of *A. naeslundii* [98].

Various techniques are used to extract phytochemicals. Extraction utilizes the selected solvents to separate medicinally active components. The conventional extraction procedure was used to retrieve required fraction and remove undesired material using solvent [99]. These items include alkaloids, glycosides, terpenoids, flavonoids, and lignones, among others. Phytochemical extraction must be thorough, efficient, simple, fast, and affordable. Soxhlet extraction and plant tissue homogenization are two procedures that have been developed throughout time [100,101].

Chemicals found in plants that are not nutritional but have protective or disease-preventing qualities are referred to as phytochemicals [102]. As a result of the antimicrobial activity of several phytochemicals generated by plants, these plants may be investigated and employed in the creation of novel antimicrobial medications. The phytochemical characterization of plant material is significant since it is related to the therapeutic effects of the plant material in question [103]. It is self-evident that various plant species would have a variety of chemical compounds of varying strength. However, these variations might include distinct types or even the same variety produced in a different place or harvested at a different time, depending on the circumstances. Different plant components, including the leaves, bark, seeds, roots, flowers, and pods, might contain a variety of active ingredients that vary from one another [104].

Kavya et al. conducted a preliminary phytochemical investigation of *Abrus pulchellus* and *Abrus precatorius* plant extracts and discovered the presence of flavonoids, alkaloids, and saponins in both plant extracts [105]. *Abrus precatorius* seeds were subjected to a phytochemical screening procedure, which indicated the presence of alkaloids, tannins, and flavonoids but not anthraquinones or glycosides [106]. A phytochemical screening of the methanol extract of *Piper betle L*. leaves was carried out, which revealed the presence of flavonoids, tannins, sterols, and phenols in the leaves of *Piper betle L*. Through the methanol extract, various compounds, such as flavonoids, tannins, steroids, alkaloids, and glycosides, as well as carbohydrates, proteins, phenols, flavonoids, and alkaloids were discovered [107].

In herbal medicine, 20% of plants are used to cure a variety of disorders, including diseases caused by pathogenic bacteria and fungus [108]. Wolde et al. found that garlic extracts have a high range of antibacterial activity against both Gram-negative and Gram-positive bacteria. The garlic extracts were also found effective against antibiotic-resistant bacteria and their toxic products. This effect was because of garlic compounds. Especially, allicin affects the growth of bacteria by partially inhibiting their DNA and protein synthesis and primarily inhibiting RNA synthesis as the main target [109]. According to Kshirsagar et al., garlic extract showed antimicrobial activity against *S. mutans* and *L. acidophilus*. During their study, they found that 18 mm to 24 mm of inhibition was observed against the above-mentioned bacteria [110]. The antibacterial, antifungal, and antiviral qualities of garlic (*Allium sativum*) make it a valuable addition to any kitchen. Garlic extracts were shown to suppress the development of harmful bacteria in aqueous, ethanol, and chloroform solutions, however, with different degrees of sensitivity to the extracts [111,112].

Garlic extract was shown to be effective against Gram-positive bacteria that are disease-causing, especially *S. mutans*. As the concentration of garlic extract increases, the width of the non-growth zone increased as well. The bacteria *L. acidophilus* was responsive to varied doses of garlic extract when cultured on blood agar medium, in a similar fashion. Once again, the width of the inhibitory zone grew in proportion to the rise in the concentration of garlic extract [113]. *Bacillus subtilis*, *S. aureus*, *Escherichia coli*, and *Salmonella typhi*, were used against the garlic extracts, and the zones of inhibition were calculated to be 29 mm, 26 mm, 46 mm, 31 mm, and 25 mm in diameter, respectively. While using streptomycin, the inhibitory zone was determined to be 35 mm, 33 mm, 29 mm, and 31 mm in width, respectively [114]. With diameter zones of inhibition ranging from 4.40 cm to 3.80 cm, garlic extract had the greatest antibacterial effect against skin pathogenic bacteria *S. aureus*, followed by *S. epidermidis* with diameter zones of inhibition ranging from 4.13 cm to 3.57 cm, and finally *Strep. pyogenes* with diameter zones of inhibition ranging from 3.40 cm to 2.67 cm. *P. aeruginosa* was found to have the smallest inhibitory zones, which were 2.32 cm–1.55 cm in diameter [115]. A 31 mm inhibitory zone has been observed for both fresh local and imported garlic extracts when used against methicillin-resistant *S. aureus* (MRSA). *S. aureus* was the most susceptible bacterium to garlic extract, with a 26 mm diameter zone of inhibition, followed by *S. enteritidis*, while *B. cereus* was found to be the most resistant bacteria to garlic extract [116]. Because of its broad range of antibacterial action, garlic extract has the potential to be used in the creation of broad-spectrum antibiotics [117].

A similar pattern was seen when comparing the zone of inhibition (9 mm) of garlic extract to that of antibiotic (ciprofloxacin) when 50% of the garlic extract was used against *Pseudomonas aeruginosa*. However, when tested against *Pseudomonas aeruginosa*, garlic extract and antibiotics were shown to be ineffective in 20% of cases [118]. The antibacterial activity of the alcoholic extract of garlic against *S. aureus* was determined to be 9 mm for the concentration of 10 mg/mL and 23 mm for the concentration of 100 mg/mL. The concentrations of 10–20 mg/mL were relatively inactive in preventing the development of *S. aureus*, the concentrations of 40–60 mg/mL were moderately inactive, and the concentrations of 80–100 mg/mL were very effective in preventing the growth of *S. aureus* [119].

According to the literature, the ethanolic extract of ginger has shown significant activity against *P. aeruginosa* and *B. subtilis* with zone of inhibition ranging from 70.4 mm at 25 mg/mL to 23 mm at 100 mg/mL, and the MIC ranges from 6.25 mg/mL to 12 mg/mL against *B. subtilis* and *C. albicans*. At low doses, the activity of the aqueous tract was very low; however, at higher concentrations, a significant amount of activity was found [120]. In addition to *Escherichia coli*, *Staphylococcus aureus*, *S. epidermidis*, *Klebsiella pneumoniae*, *Salmonella typhi*, *S. typhimurium*, *Pseudomonas aeruginosa*, *Proteus* sp., *Bacillus cereus*, *Bacillus subtilis*, *Bacillus megaterium*, *Streptococcus faecalis*, *Enterococcus faecalis*, *Pseudomonas aeruginosa*, and *Proteus* sp., ginger shows antibacterial properties against various other Gram-positive and the Gram-negative bacteria [121]. The antimicrobial effectiveness of fresh, natural, and commercial dried ginger extracts has been evaluated against seven local clinical bacterial isolates using the agar disc diffusion technique. The results demonstrated that the chloroform and diethyl ether extracts of ginger, with the exception of *P. aeruginosa* and *E. coli*, displayed a more substantial inhibitory zone of the pathogens examined [122].

Neem extracts are high in antibacterial components, making them potentially effective in the control of certain foodborne pathogens and other spoilage organisms. Neem leaf extracts have antibacterial characteristics, and the extract exhibited considerably higher zones of inhibition than 3% sodium hypochlorite, indicating that the extract has antimicrobial capabilities [123].

Bacterial strains were evaluated based on the width of the growth inhibition zone achieved by using concentrations of 20 mg/mL and 40 mg/mL Ocimum methanol extract, respectively. By using this procedure, the extracts can diffuse more effectively into the medium, increasing interaction with the organisms. The antibacterial activity of tulsi extracts was tested against four pathogenic species: *Escherichia coli*, *Staphylococcus aureus*, *Aeromonas hydrophila*, and *Enterococcus faecalis*. The results showed that the extracts were effective against all four pathogenic organisms. The findings revealed that the Ocimum extracts at final concentrations of 40 mg/mL were effective. In contrast, the final concentration of 40 mg/mL methanol extract of the antioxidant was higher in the liver than in the muscle systems when exposed to oxidative stress [124].

Variya et al. indicated that both the aqueous and methanol extracts of amla were effective against a variety of pathogenic organisms, including *Klebsiella pneumoniae*, *E. cloacae*, and *E. coli* [125]. The antibacterial activity of amla has been seen to vary when tested against Gram-positive and Gram-negative pathogenic bacterial species, including *Bacillus subtilis*, *Staphylococcus aureus*, *Escherichia coli*, and *Pseudomonas aeruginosa*, which were successfully treated using silver nanoparticles created by green synthesis using the fruits and leaves of amla [126].

Ramanuj et al. observed the bacteriostatic effect of Emblica officinalis seed fractions against *Acinetobacter baumannii* [127]. Amla’s antiplasmodial effect has been studied in vivo and in vitro. Both chloroquine-resistant and chloroquine-sensitive isolates of *P. falciparum* were found to be resistant to amla leaves extract in ethyl acetate. The antimalarial effect was shown against both chloroquine-sensitive and chloroquine-resistant plasmodium species [128]. The ethanolic extract, chloroform, acetone, and aqueous portions of amla fruits exhibited promising antimicrobial activity against a variety of bacterial species, including *Escherichia coli*, *Proteus* sp., *Salmonella paratyphi*, *Pseudomonas* sp., *Staphylococcus aureus*, *Klebsiella* sp., *Bacillus* sp., and *Salmonella* [128]. A potential inhibitory effect against *Candida galbrata* and *Cryptococcus neoformans* was seen in the ethanolic fraction of amla; however, there was no cytotoxic activity observed in the vero cell line [125]. It was discovered that the chloroform soluble fraction of the amla methanol extract exhibited considerable and promising antibacterial activity, as well as significant cytotoxicity, when tested against a variety of pathogenic Gram-positive and Gram-negative bacteria [129].

Aloe vera plant extracts possess antimicrobial properties that kill or inhibit the development of microorganisms (including bacteria (antibacterial activity)), fungi (antifungal activity), and viruses (antiviral activity). Fruit rot is a significant factor impacting the postharvest quality of fresh food after it has been harvested. A number of previous research have shown that the application of aloe vera gel as an edible coating has beneficial effects on the prevention of fruit deterioration and microbiological spoiling [130,131]. Aloe vera gel has an inhibiting impact on the growth of mycelium *(Penicillium digitatum and Aspergillus niger*) [132]. The rate of mycelium development increased with the concentration of gel used. Aloe vera gel at a concentration of 500 mL/l was shown to suppress the growth of *P. digitatum* and *A. niger* at 100% and 64%, respectively [133].

Dental caries is a multifactorial human disease found all over the world. It is considered one of the main problems of public health. During the last two decades, dental caries remains a severe global concern. There is a significant correlation between having poor dental health and an increased likelihood of developing systemic diseases. The results of our study are in line with the previous literature, where our study results are supported by the previous studies; herbal plants have been used against the varieties of microbes. Bacterial antibiotics are frequently utilized in the treatment of dental caries and other dental-related disorders, both therapeutically and prophylactically. Further research studies on the topic are needed and necessary to verify the results obtained in this study [85]. 

## 5. Conclusions

The results obtained during this study showed that selected herbal plants have antimicrobial activities against the oral microbes obtained from patients with dental caries. The current research study concluded that the disease prevalence was higher in males as compared with females, more prevalent in middle-aged patients, and toothache was the most common symptom of dental caries. Seven cariogenic bacterial strains were isolated, among which *S. mutans* and *S. sobrinus* were found as the most abundant bacterial strains. Among synthetic antibiotics, ciprofloxacin and levofloxacin were the most effective against all isolated cariogenic microorganisms. Comparatively, among plant extract antimicrobial activity, ginger and amla exhibited the maximum antibiotic activity against all isolated strains, making them the most suitable alternatives to synthetic antibiotics for treating dental diseases. Plant extract antimicrobial activity against cariogenic bacterial strains is the most preferable choice in the future to avoid upcoming bacterial resistance to antibiotics.

## Figures and Tables

**Table 1:** Preparation of plant extracts according to the following protocols.

Antimicrobial Plants	Preparation of Plant Extracts According to the Following Protocols		
	Aqueous Extract	Methanolic Extract	Ethanolic Extract
Garlic	[34]	[43]	[44]
Ginger	[45]	[46,47]	[48]
Neem	[49]	[50]	[51]
Tulsi	[52]	[53]	[54,55]
Amla	[56]	[56]	[57]
Aloe vera	[58]	[59]	[60]

**Table 2:** List of bacterial growth media with modification.

Bacterial Strains	Growth Media	Reference
*S. sobrinus*	Ultrafiltered tryptone-yeast extract (UTE) broth with the addition of 1% glucose	[65]
*S. mutans*		
*A. naeslundii*	Trypticase soy glucose agar	[66]
*L. acidophilus*	Carbohydrate-free MRS medium	[67,68]
*E. nodatum*	BAP and BHIA	[69]
*P. acidifaciens*	BHIA supplemented with 0.5% yeast extract and 0.03% cysteine HCl (BYC broth)	[70]
*T. pallidum*	BAP	[71]

**Table 3:** Colony characteristics of isolates on different isolation and selective media. Note: MSB agar, Mitis Salivarius Bacitracin agar; BHI agar, Brain Heart Infusion agar; SB-20M, sucrose-bacitracin selective medium.

Isolate	Colony Characteristics on Different Isolation and Selective Media						
	DKF 001	DKF 002	DKF 003	DKF 004	DKF 005	DKF 006	DKF 007
**Blood agar**	Grey to black small, round, mucoid colonies with hemolysis	White or grey, circular or irregular	White, rough, dry pinpoint colonies	Small to medium grey-colored colonies	Small, convex colonies having entire margins	Circular, entire margins, dome-shaped surface, white to pale cream color	Imbedded, round colonies, non-hemolytic, 0.3–0.6 mm
**BHI agar**	Small, pale, opaque circular, mucoid colonies	Small, off-white to pale, opaque, circular, mucoid colonies	White, cream to pink-colored colonies		White to off-white colonies, embedded in agar		
**MSB agar**	Smooth, raised, convex, pale blue colonies	Rough, raised, convex, undulate, opaque, pale blue colonies					
**SB-20M agar**	Circular and opaque milky white, exhibiting polysaccharide drops	Granular surface colonies resembling ground glass, polysaccharide drop on the surface					

DKF 001, DKF 002, DKF 003, DKF 004, DKF 005, and DKF 006 were notified as Gram-positive, non-spore-forming, and non-motile. DKF 001 showed purple-colored colonies, spherical shape, appear in pairs or chains, and were non-spore-forming and non-motile. The culture conditions depicted that this strain was anaerobic. DKF 002 retained a purple stain and appeared as cocci, arranged in pairs or short chains. DKF 003 were rod-shaped. DKF 004 were rod-shaped coccobacilli, having clubbed ends with branching. DKF 005 were rod-shaped with branches. The growth conditions depicted it as an anaerobe. DKF 006 were approximately 2.6 μm long and 0.75 μm wide and exhibited pigment-producing ability. DKF007 were Gram-negative spirochete, microaerophilic, thin, corkscrew-shaped, 6–20 μm long, 0.1–0.2 μm wide, and tightly coiled.

**Table 4:** Enzyme activity analysis of isolates from oral microbial flora. NEA, no enzymatic activity; ONPG, O-nitrophenyl-beta-D-galactopyranoside; MR, methyl red; VP, Voges-proskauer; PAD, phenylalanine deaminase.

Isolate Number	Enzyme Activity Tests																
	Hydrolysis									Decarboxylation							
	Esculin	Arginine	Urease	Starch	ONPG	Casein	Gelatin	Lysine	Ornithine	Nitrate Reduction	Citrate	Catalase	Indole	MR	VP	H_2_S	PAD
DKF 001	+	-	-	+	-	NEA	-	NEA		-	+	-	-	-	+	-	NEA
DKF 002	+	-	-		-	-	-			-	-	+		-	+	-	
DKF 003	+	-	+	+	+	-	-			+	NEA	-	-	NEA	NEA	+	+
DKF 004	+	-	-	+	+	-	-	-	+	-	-	-	-	-	+	-	-
DKF 005	-	-	-	-	-	NEA	-	NEA	NEA	-	NEA	-	-	+	-	-	NEA
DKF 006	-	-	-	NEA	+		-			-	NEA	+	-	NEA		-	
DKF 007	NEA																

**Table 5:** Fermentation of different sugars by isolates from oral microbial flora.

Isolate	Sugar Fermentation Analysis														
	Glucose	Lactose	Mannitol	Mannose	Sucrose	Sorbitol	Inulin	Raffinose	Maltose	Trehalose	Xylose	Arabinose	Rhamnose	Dulcitol	Fructose
DKF 001	+	+	+	+	+	-	-	+	+	+	-	-	-	-	
DKF 002	+	+	+	+	+	+	+	+	+	+	-	-	-	-	+
DKF 003	+	+	-	+	+	+	+	+	+	+	-	-	-	-	+
DKF 004	+	+	-	+	+	-	-	+	+	-	+	+	+	-	+
DKF 005	+	+	-	-	+	-	+	-	+	-	-	-	-	+	-
DKF 006	+	+	+	+	+	-	+	+	+	-	+	-	+	-	+
DKF 007	-	+	-	-	+	+	-	-	+	+	+	-	-	+	-

**Table 6:** Physiological test of isolates from oral microbial flora.

Isolate Number	Growth in Different Concentrations of NaCl			
	2%	5%	7%	10%
DKF 001	+	-	-	-
DKF 002	+	+	-	-
DKF 003	+	+	-	-
DKF 004	+	+	-	-
DKF 005	+	+	+	+
DKF 006	+	+	-	-
DKF 007	+	-	-	-

**Table 7:** Physical properties of aquatic, methanolic, and ethanolic extracts.

Solvent Used	Physical Characteristics	Garlic	Ginger	Neem	Tulsi	Amla	Aloe Vera
**Water**	Color	Light yellow	Yellowish pink	Dark green	Dark brown	Yellowish green	Faint green
	Odor	Pungent	Pungent	Organic	Aromatic	Aromatic	Organic
	Consistency	Off white liquid	Pale yellowish liquid	Grassy greenish liquid	Light brownish liquid	Yellowish green liquid	Semi solid sticky
**Methanol**	Color	Faint yellow	Yellowish pink	Dark green	Dark brown	Brownish black	Faint green
	Odor	Pungent	Pungent	Agreeable	Aromatic	Aromatic	Organic
	Consistency	Light yellowish powder	Yellowish powder	Olive green liquid	Brownish crystalline solid	Light green solid	Greenish sticky solid
**Ethanol**	Color	Light yellow	Yellowish pink	Olive green	Dark brown	Brownish black	Faint green
	Odor	Pungent	Pungent	Organic	Aromatic	Aromatic	Agreeable
	Consistency	Yellowish sticky liquid	Yellowish powder	Dried Light green	Brownish fine powder	Olive green liquid	Greenish sticky semi solid

**Table 8:** The percentage yield of medicinal plant extracts using different solvents.

Solvent Used	Physical Characteristics	Garlic	Ginger	Neem	Tulsi	Amla	Aloe Vera
Water	Weight of dry powder (g)	15	15	15	15	15	15
	Weight of extract (g)	3.91	8.43	4.87	7.1	6.47	7.69
	% Yield	26.07	56.23	32.47	47.33	43.14	51.27
Methanol	Weight of dry powder (g)	15	15	15	15	15	15
	Weight of extract (g)	5.1	9.44	5.32	3.97	4.88	7.23
	% Yield	34	62.93	35.47	26.47	32.53	48.2
Ethanol	Weight of dry powder (g)	15	15	15	15	15	15
	Weight of extract (g)	4.94	6.54	6.13	3.26	5.17	6.98
	% Yield	32.93	43.6	40.87	21.73	34.47	46.53

**Table 9:** Phytochemical analysis of extracts.

Plant	Extract	Alk	Sap	Ta	Fl	C. Gly	F and F.O	Carb.	St and Ter	Pro	A.A
Garlic	Aqueous	-	+	-	+	+	+	+	+	+	+
	Methanol	+	+	+	-	+	-	+	+	-	-
	Ethanol	+	+	-	+	+	+	+	+	-	-
Ginger	Aqueous	-	-	-	-	-	-	+	+	-	
	Methanol	-	+	+	+	+	+	+	+	-	-
	Ethanol	+	+	+	+	-	+	+	+	+	-
Neem	Aqueous	+	+	-	+	-	-	-	+	-	+
	Methanol	+	-	+	-	+	-	+	+	+	+
	Ethanol	-	+	+	+	+	+	+	-	+	-
Tulsi	Aqueous	-	+	+	+	-	+	-	+	-	-
	Methanol	-	+	-	-	+	+	-	-	+	+
	Ethanol	+	-	+	+	+	+	+	-	+	+
Amla	Aqueous	+	+	+	-	-	-	+	+	+	+
	Methanol	+	+	-	-	-	+	-	-	-	-
	Ethanol	+	-	+	+	+	+	-	+	+	+
Aloe vera	Aqueous	+	+	+	-	+	-	+	-	-	-
	Methanol	-	+	+	+	-	+	-	-	+	+
	Ethanol	-	-	-	+	+	+	+	+	-	-

**Note**: -, negative; +, positive; Alk, alkaloids; Sap, saponins; Ta, tannins; Fl, flavonoids; C. Gly, cardiac glycosides; F and F.O, fats and fixed oils; Carb, carbohydrates; St, steroids; Ter, terpinoids; Pro, proteins; A.A, amino acids.

**Table 10:** Zone of inhibition selected medicinal plants against isolated bacterial strains.

Medium	Strength	Zone of Inhibition for *S. sobrinus*					
		Garlic	Ginger	Neem	Tulsi	Amla	Aloe Vera
Aqueous	25 mg/mL	11.03	8.39	12.13	8.08	17.52	9.31
	50 mg/mL	13.91	10.93	13.86	9.34	18.04	10.93
	75 mg/mL	15.03	12.61	14.17	10.55	18.69	11.71
	100 mg/mL	18.59	15.61	14.76	10.54	20.01	11.91
Methanolic	25 mg/mL	9.67	15.87	11.32	10.88	7.54	11.95
	50 mg/mL	9.89	17.60	11.72	12.67	8.23	12.49
	75 mg/mL	10.12	19.66	12.80	14.19	9.29	13.89
	100 mg/mL	12.31	20.94	11.86	14.83	9.59	14.71
Ethanolic	25 mg/mL	8.74	21.46	12.88	11.49	11.53	8.72
	50 mg/mL	9.87	22.07	13.09	12.57	12.41	9.79
	75 mg/mL	11.23	22.36	13.41	13.01	12.93	11.07
	100 mg/mL	12.59	24.91	14.49	13.98	13.05	11.69
Zone of Inhibition for *S. mutans*							
Aqueous	25 mg/mL	10.13	8.05	13.43	12.90	16.42	7.28
	50 mg/mL	11.21	9.56	13.86	13.49	16.64	7.93
	75 mg/mL	11.35	10.41	13.97	13.74	17.09	8.61
	100 mg/mL	12.59	11.11	14.46	14.14	18.01	8.91
Methanolic	25 mg/mL	13.76	12.67	15.12	13.89	6.52	12.95
	50 mg/mL	14.89	13.64	15.51	14.37	7.21	12.49
	75 mg/mL	14.92	13.96	15.98	14.69	8.49	13.19
	100 mg/mL	15.21	14.94	16.86	14.97	8.79	13.71
Ethanolic	25 mg/mL	9.72	19.86	14.88	13.19	13.49	12.72
	50 mg/mL	10.17	20.57	15.12	13.67	13.88	12.91
	75 mg/mL	11.35	21.16	15.43	13.91	13.93	13.27
	100 mg/mL	13.19	21.95	15.79	13.95	14.15	13.59
Zone of Inhibition for *A. naeslundii*							
Aqueous	25 mg/mL	8.05	16.42	8.05	13.43	6.52	12.67
	50 mg/mL	10.13	16.64	9.56	13.86	7.21	12.9
	75 mg/mL	11.21	17.09	10.41	13.97	8.49	12.95
	100 mg/mL	11.35	13.49	11.11	14.12	8.79	13.27
Methanolic	25 mg/mL	9.56	13.88	12.67	15.51	7.28	13.89
	50 mg/mL	10.41	13.93	13.64	15.98	7.93	14.37
	75 mg/mL	13.43	14.15	13.96	16.86	8.61	14.69
	100 mg/mL	13.86	14.89	13.74	16.93	8.91	14.97
Ethanolic	25 mg/mL	12.90	10.17	6.52	12.72	9.72	14.88
	50 mg/mL	13.49	11.35	7.21	12.91	10.17	15.12
	75 mg/mL	13.74	13.19	8.49	13.27	11.35	15.43
	100 mg/mL	13.97	14.92	9.72	13.59	13.19	15.79
Zone of Inhibition for *L. acidophilus*							
Aqueous	25 mg/mL	12.67	16.42	13.43	8.15	8.05	6.50
	50 mg/mL	12.90	16.64	13.86	9.13	9.56	7.21
	75 mg/mL	12.95	17.09	13.97	10.21	10.41	8.49
	100 mg/mL	13.27	17.49	14.12	10.35	11.11	8.79
Methanolic	25 mg/mL	13.89	13.88	15.51	9.56	12.67	7.28
	50 mg/mL	14.37	13.93	15.98	10.41	13.64	7.93
	75 mg/mL	14.69	14.15	16.86	13.43	13.96	8.61
	100 mg/mL	14.97	14.89	16.93	13.86	13.74	8.91
Ethanolic	25 mg/mL	14.88	10.17	12.72	12.9	6.52	9.72
	50 mg/mL	15.12	11.35	12.91	13.49	7.21	10.17
	75 mg/mL	15.43	13.19	13.27	13.74	8.49	11.35
	100 mg/mL	15.79	14.92	13.59	13.97	9.72	13.19
Zone of Inhibition for *E. nodatum*							
Aqueous	25 mg/mL	16.54	13.56	12.78	8.15	9.35	6.60
	50 mg/mL	16.72	13.92	12.96	9.61	10.13	7.34
	75 mg/mL	17.18	14.01	13.02	10.48	11.21	8.55
	100 mg/mL	13.56	14.16	13.37	11.15	11.35	8.88
Methanolic	25 mg/mL	13.96	15.62	13.97	12.71	9.56	7.33
	50 mg/mL	14.03	16.07	14.49	13.74	10.41	8.02
	75 mg/mL	14.28	16.94	14.81	14.00	13.43	8.69
	100 mg/mL	14.97	17.00	15.03	13.80	13.86	9.04
Ethanolic	25 mg/mL	10.30	12.83	15.00	6.56	12.9	9.79
	50 mg/mL	11.43	13.04	15.25	7.29	13.49	10.30
	75 mg/mL	13.28	13.4	15.51	8.61	13.74	11.44
	100 mg/mL	15.03	13.72	15.87	9.79	13.97	13.31
Zone of Inhibition for *P. acidifaciens*							
Aqueous	25 mg/mL	12.02	13.47	16.39	8.25	6.52	6.59
	50 mg/mL	12.58	13.91	16.62	10.73	7.22	7.25
	75 mg/mL	12.94	14.02	17.11	11.01	8.51	8.50
	100 mg/mL	13.30	14.17	17.50	11.35	9.75	8.80
Methanolic	25 mg/mL	13.90	15.59	13.88	9.56	8.10	7.32
	50 mg/mL	14.42	16.07	13.93	10.41	9.58	7.96
	75 mg/mL	14.77	16.92	14.20	11.43	10.43	8.70
	100 mg/mL	14.99	16.93	14.92	13.86	11.12	8.91
Ethanolic	25 mg/mL	14.91	12.73	10.18	12.90	12.70	9.72
	50 mg/mL	15.18	12.93	11.35	13.49	13.64	10.25
	75 mg/mL	15.43	13.27	13.15	13.74	13.96	11.44
	100 mg/mL	15.81	13.67	14.89	13.97	13.82	13.2
Zone of Inhibition for *T. pallidum*							
Aqueous	25 mg/mL	10.15	12.9	12.8	12.67	6.56	8.09
	50 mg/mL	11.37	13.49	12.98	12.91	7.21	9.56
	75 mg/mL	13.23	13.74	13.27	12.97	8.57	10.42
	100 mg/mL	14.89	13.97	13.67	13.29	8.81	11.12
Methanolic	25 mg/mL	13.90	8.05	13.44	14.94	7.33	12.69
	50 mg/mL	13.96	10.13	13.94	15.14	8.02	13.67
	75 mg/mL	14.11	11.21	14.05	15.47	8.70	13.98
	100 mg/mL	14.91	11.35	14.19	15.87	9.00	13.74
Ethanolic	25 mg/mL	16.47	9.56	15.6	13.95	9.73	6.54
	50 mg/mL	16.64	10.41	16.06	14.45	10.23	7.22
	75 mg/mL	17.09	13.43	16.87	14.76	11.36	8.51
	100 mg/mL	17.53	13.86	17.02	15.05	13.22	9.74

**Table 11:** Antibacterial activity by synthetic antibiotics.

Name of Agent (25 mg/mL)	*S. sobrinus*	*S. mutans*	*A. naeslundii*	*L. acidophilus*	*E. nodatum*	*P. acidifaciens*	*T. pallidum*
Penicillin	27.13	23.35	11.4	13.1	22.5	7.3	7.8
Amoxicillin	19.22	21.05	9.1	12.3	15.4	8.5	7.8
Erythromycin	14.31	15.03	12.5	14.6	16.3	8.9	8.4
Clindamycin	25.36	23.5	19.6	21.3	22.4	8.2	8.7
Metronidazole	14.57	17.61	17.3	18.8	15.9	6.5	8.2
Doxycycline	19.38	21.04	19.3	20.3	22.6	7.9	7.3
Ceftazidime	22.47	23.25	21.4	19.8	19.3	12.1	9.1
Levofloxacin	29.98	25.55	22.7	23.3	28.1	14.3	10.3
Ciprofloxacin	35.44	28.67	29.8	27.4	29.5	14.3	10.6
DMSO (10%)	Negative control						

## Data Availability

The data presented in this study are available in the Supplementary Materials.

## Supplementary Materials

The following supporting information can be downloaded at: https://www.mdpi.com/article/10.3390/microorganisms11081952/s1, Table S1: Primers used in PCR amplification of conserved region in 16S rDNA gene for bacterial strains; Table S2: ANOVA for antibacterial activity of herbal extracts for *S. sobrinus*; Table S3: ANOVA for antibacterial activity of herbal extracts for *S. mutans*; Table S4: ANOVA for antibacterial activity of herbal extracts for *A. naeslundii*; Table S5: ANOVA for antibacterial activity of herbal extracts for *L. acidophilus*; Table S6: ANOVA for antibacterial activity of herbal extracts for *E. nodatum*; Table S7: ANOVA for antibacterial activity of herbal extracts for *P. acidifaciens*; Table S8: ANOVA for antibacterial activity of herbal extracts for *T. pallidum*. References [134,135,136,137,138] are cited in the supplementary materials.

## References

[1] Souza J.C., Henriques M., Teughels W., Ponthiaux P., Celis J.-P., Rocha L.A. (2015). Wear and corrosion interactions on titanium in oral environment: Literature review. J. Bio-Tribo-Corros..

[2] Xu W., Bronlund J., Potgieter J., Foster K., Röhrle O., Pullan A., Kieser J. (2008). Review of the human masticatory system and masticatory robotics. Mech. Mach. Theory.

[3] Whitlock J.A., Richman J.M. (2013). Biology of tooth replacement in amniotes. Int. J. Oral. Sci..

[4] Tjäderhane L., Carrilho M.R., Breschi L., Tay F.R., Pashley D.H. (2009). Dentin basic structure and composition—An overview. Endod. Top..

[5] Robinson C., Brookes S., Kirkham J., Bonass W., Shore R. (1996). Crystal growth in dental enamel: The role of amelogenins and albumin. Adv. Dent. Res..

[6] Kalra D.D., Kalra R.D., Kini P.V., Prabhu C.A. (2014). Nonfluoride remineralization: An evidence-based review of contemporary technologies. J. Dent. Allied Sci..

[7] de Souza Leao T.S., Zanoni A.V., Franzon R., Tomasi G.H., Conzatti L.P., Marrone L.C.P., Reynolds M.A., Gomes M.S. (2021). Number of teeth is independently associated with ischemic stroke: A case-control study. J. Clin. Neurosci..

[8] Bik E.M., Long C.D., Armitage G.C., Loomer P., Emerson J., Mongodin E.F., Nelson K.E., Gill S.R., Fraser-Liggett C.M., Relman D.A. (2010). Bacterial diversity in the oral cavity of 10 healthy individuals. ISME J..

[9] Gayathri D., Rashmi B. (2016). Microbial Diversity in Different Organs of Human System. J. Food Microbiol..

[10] Seymour G.J., Ford P.J., Cullinan M.P., Leishman S., Yamazaki K. (2007). Relationship between periodontal infections and systemic disease. Clin. Microbiol. Infect..

[11] Joshipura K.J., Rimm E., Douglass C., Trichopoulos D., Ascherio A., Willett W. (1996). Poor oral health and coronary heart disease. J. Dent. Res..

[12] Beck J.D., Offenbacher S. (2005). Systemic effects of periodontitis: Epidemiology of periodontal disease and cardiovascular disease. J. Periodontol..

[13] Irshad M., Alam M.K., Alawneh A., Alhadi M.A., Alhadi A.A., Almunajem Y.S., Alanezi F.F., Al Sagoor S.A., Bajawi A.M., Alfawzan A.A. (2020). Characterization and antimicrobial susceptibility of pathogens associated with periodontal abscess. Antibiotics.

[14] Bai H., He W., Chau J.H., Zheng Z., Kwok R.T., Lam J.W., Tang B.Z. (2021). AIEgens for microbial detection and antimicrobial therapy. Biomaterials.

[15] Franco-Duarte R., Černáková L., Kadam S., Kaushik K.S., Salehi B., Bevilacqua A., Corbo M.R., Antolak H., Dybka-Stępień K., Leszczewicz M. (2019). Advances in chemical and biological methods to identify microorganisms—From past to present. Microorganisms.

[16] Baums I.B., Baker A.C., Davies S.W., Grottoli A.G., Kenkel C.D., Kitchen S.A., Kuffner I.B., LaJeunesse T.C., Matz M.V., Miller M.W. (2019). Considerations for maximizing the adaptive potential of restored coral populations in the western Atlantic. Ecol. Appl..

[17] Wu S.Y., Yau H.S., Yu M.Y., Tsang H.F., Chan L.W.C., Cho W.C.S., Shing Yu A.C., Yuen Yim A.K., Li M.J., Wong Y.K.E. (2020). The diagnostic methods in the COVID-19 pandemic, today and in the future. Expert. Rev. Mol. Diagn..

[18] Perrone A., Giovino A., Benny J., Martinelli F. (2020). Advanced glycation end products (AGEs): Biochemistry, signaling, analytical methods, and epigenetic effects. Oxidative Med. Cell. Longev..

[19] Klein E.Y., Van Boeckel T.P., Martinez E.M., Pant S., Gandra S., Levin S.A., Goossens H., Laxminarayan R. (2018). Global increase and geographic convergence in antibiotic consumption between 2000 and 2015. Proc. Natl. Acad. Sci. USA.

[20] Gajdács M. (2019). The continuing threat of methicillin-resistant Staphylococcus aureus. Antibiotics.

[21] Saroya A.S., Singh J., Saroya A.S., Singh J. (2018). Introduction to Herbal Medicine. Pharmacother. Potential. Nat. Prod. Neurol. Disord..

[22] Aneja K.R., Joshi R., Sharma C. (2010). In vitro antimicrobial activity of Sapindus mukorossi and Emblica officinalis against dental caries pathogens. Ethnobot. Leafl..

[23] Inchingolo A.D., Malcangi G., Semjonova A., Inchingolo A.M., Patano A., Coloccia G., Ceci S., Marinelli G., Di Pede C., Ciocia A.M. (2022). Oralbiotica/Oralbiotics: The Impact of Oral Microbiota on Dental Health and Demineralization: A Systematic Review of the Literature. Children.

[24] Cappuccino N., Mackay R., Eisner C. (2002). Spread of the invasive alien vine Vincetoxicum rossicum: Tradeoffs between seed dispersability and seed quality. Am. Midl. Nat..

[25] Liu Y.-L., Nascimento M., Burne R.A. (2012). Progress toward understanding the contribution of alkali generation in dental biofilms to inhibition of dental caries. Int. J. Oral. Sci..

[26] Velsko I.M., Fellows Yates J.A., Aron F., Hagan R.W., Frantz L.A., Loe L., Martinez J.B.R., Chaves E., Gosden C., Larson G. (2019). Microbial differences between dental plaque and historic dental calculus are related to oral biofilm maturation stage. Microbiome.

[27] Sridhara P.B., Dharmashekara C., Srinivasa C., Shivamallu C., Kollur S.P., Gopinath S., Syed A., Patil S.S., Prasad A., Salamun D. (2021). Isolation, Characterization, and Optimization of Protease-Producing Bacterium Bacillus thuringiensis from Paddy Field Soil. Pharmacogn. Res..

[28] Bahuguna A., Joe A.-r., Kumar V., Lee J.S., Kim S.-Y., Moon J.-Y., Cho S.-K., Cho H., Kim M. (2020). Study on the identification methods for effective microorganisms in commercially available organic agriculture materials. Microorganisms.

[29] Mulaw G., Sisay Tessema T., Muleta D., Tesfaye A. (2019). In vitro evaluation of probiotic properties of lactic acid bacteria isolated from some traditionally fermented Ethiopian food products. Int. J. Microbiol..

[30] Hoppe H.-G. (2018). Use of fluorogenic model substrates for extracellular enzyme activity (EEA) measurement of bacteria. Handbook of Methods in Aquatic Microbial Ecology.

[31] Kootallur B., Thangavelu C., Mani M. (2011). Bacterial identification in the diagnostic laboratory: How much is enough?. Indian. J. Med. Microbiol..

[32] Wright M.H., Adelskov J., Greene A.C. (2017). Bacterial DNA extraction using individual enzymes and phenol/chloroform separation. J. Microbiol. Biol. Educ..

[33] Wilson K. (2001). Preparation of genomic DNA from bacteria. Curr. Protoc. Mol. Biol..

[34] Eja M.E., Asikong B.E., Abriba C., Arikpo G.E., Anwan E.E., Enyi-Idoh K.H. (2007). A comparative assessment of the antimicrobial effects of garlic (*Allium sativum*) and antibiotics on diarrheagenic organisms. Southeast. Asian J. Trop. Med. Public. Health.

[35] Akintobi O., Onoh C., Ogele J., Idowu A., Ojo O., Okonko I. (2013). Antimicrobial activity of *Zingiber officinale* (ginger) extract against some selected pathogenic bacteria. Nat. Sci..

[36] Giriraju A., Yunus G. (2013). Assessment of antimicrobial potential of 10% ginger extract against *Streptococcus mutans*, *Candida albicans*, and *Enterococcus faecalis*: An in vitro study. Indian. J. Dent. Res..

[37] Lloyd A.C., Menon T., Umamaheshwari K. (2005). Anticandidal activity of Azadirachta indica. Indian. J. Pharmacol..

[38] Coventry E., Allan E.J. (2001). Microbiological and chemical analysis of neem (*Azadirachta indica*) extracts: New data on antimicrobial activity. Phytoparasitica.

[39] Agarwal P., Nagesh L. (2010). Evaluation of the antimicrobial activity of various concentrations of Tulsi (*Ocimum sanctum*) extract against *Streptococcus mutans*: An in vitro study. Indian. J. Dent. Res..

[40] Al-Gbouri N., Hamzah A. (2018). Evaluation of *Phyllanthus emblica* extract as antibacterial and antibiofilm against biofilm formation bacteria. Iraqi J. Agric. Sci..

[41] Fani M., Kohanteb J. (2012). Inhibitory activity of Aloe vera gel on some clinically isolated cariogenic and periodontopathic bacteria. J. Oral. Sci..

[42] Jain S., Rathod N., Nagi R., Sur J., Laheji A., Gupta N., Agrawal P., Prasad S. (2016). Antibacterial effect of Aloe vera gel against oral pathogens: An in-vitro study. J. Clin. Diagn. Res. JCDR.

[43] Bontempo P., Stiuso P., Lama S., Napolitano A., Piacente S., Altucci L., Molinari A.M., De Masi L., Rigano D. (2021). Metabolite profile and in vitro beneficial effects of black garlic (*Allium sativum* L.) polar extract. Nutrients.

[44] Liu J., Mahmood M.S., Abbas R.Z., Dillawar A., Nawaz Z., Luqman M., Abbas A., Rafique A. (2021). Therapeutic appraisal of ethanolic and aqueous extracts of clove (*Syzygium aromaticum*) and garlic (*Allium sativum*) as antimicrobial agent. Pak. J. Agric. Sci..

[45] Berkovich L., Earon G., Ron I., Rimmon A., Vexler A., Lev-Ari S. (2013). *Moringa oleifera* aqueous leaf extract down-regulates nuclear factor-kappaB and increases cytotoxic effect of chemotherapy in pancreatic cancer cells. BMC Complement. Altern. Med..

[46] Handa S. (2008). An overview of extraction techniques for medicinal and aromatic plants. Extr. Technol. Med. Aromat. Plants.

[47] Iotsor B.I., Iseghohi F., Oladoja O.E., Raji O., Yusuf Z., Oyewole O.A. (2019). Antimicrobial activities of garlic and ginger extracts on some clinical isolates. Int. J. Biotechnol..

[48] Beristain-Bauza S.D.C., Hernández-Carranza P., Cid-Pérez T.S., Ávila-Sosa R., Ruiz-López I.I., Ochoa-Velasco C.E. (2019). Antimicrobial activity of ginger (*Zingiber officinale*) and its application in food products. Food Rev. Int..

[49] Prabagar S., Nanthakumar J., Prabagar J., Thuraisingam S. (2020). Antimicrobial activity of Azadirachta Indica (neem) leaves and stem bark aqueous extracts. SPC J. Plant Sci..

[50] Kumar V., Chakraborty A., Kaur M., Pandey S., Jena M.K. (2018). Comparative study on antimicrobial activity of tulsi (*Ocimum sanctum*) and neem (*Azadirachta indica*) methanol extract. Asian J. Pharm. Clin. Res..

[51] Sakha H., Hora R., Shrestha S., Acharya S., Dhakal D., Thapaliya S., Prajapati K. (2018). Antimicrobial activity of ethanolic extract of medicinal plants against human pathogenic bacteria. Tribhuvan Univ. J. Microbiol..

[52] Senthamil Pandian C., Radhakrishnan L., Karunakaran R., Gopala Krishna Murthy T., Appa Rao V., Shamsudeen P. (2021). Antimicrobial activity of selected phytobiotics individually and in combination against gram positive and gram negative bacteria. J. Entomol. Zool. Stud..

[53] Dixit A., Gulati B., Sharma G., Bhatia G., Priya R., Bhattacharya S. (2021). Evaluation of phytochemical and antimicrobial activity of *Ocimum* spp.. Integr. Food Nutr. Metab..

[54] Mallikarjun S., Rao A., Rajesh G., Shenoy R., Pai M. (2016). Antimicrobial efficacy of Tulsi leaf (*Ocimum sanctum*) extract on periodontal pathogens: An in vitro study. J. Indian. Soc. Periodontol..

[55] Mittal R., Kumar R., Chahal H. (2018). Antimicrobial activity of Ocimum sanctum leaves extracts and oil. J. Drug Deliv. Ther..

[56] Gautam A., Shukla S. (2017). Emblica officinalis (Amla) leaf extract potentiates antibacterial activity of some antibiotics. J. Pharmacogn. Phytochem..

[57] Jahan N., Akter S. (2015). Assessment of the antimicrobial activity of the ethanolic extract of *Phyllanthus emblica* in combination with different classes of antibiotics against single and multi-drug resistant strains. J. Pharmacogn. Phytochem..

[58] Durrani F., Ullah S., Chand N., Durrani Z., Akhtar S. (2008). Using aqueous extract of aloe gel as anticoccidial and immunostimulant agent in broiler production. Sarhad J. Agric..

[59] Das P., Srivastav A.K. (2015). Phytochemical extraction and characterization of the leaves of *Aloe vera barbadensis* for its anti-bacterial and anti-oxidant activity. Int. J. Sci. Res..

[60] Ramachandraiahgari R.M.Y., Somesula S.R., Adi P.J., Mannur I.S., Enamala M., Matcha B. (2012). Protective role of ethanolic extract of Aloe vera antioxidant properties on liver and kidney of streptozotocin-induced diabetic rats. Dig. J. Nanomater. Biostructures.

[61] Raaman N. (2006). Phytochemical techniques.

[62] Shah P., Modi H., Shukla M., Lahiri S.K. (2014). Preliminary phytochemical analysis and antibacterial activity of *Ganoderma lucidum* collected from Dang District of Gujarat, India. Int. J. Curr. Microbiol. App Sci..

[63] Radulescu C., Olteanu R.L., Stihi C., Florescu M., Lazurca D., Dulama I.D., Stirbescu R.M., Teodorescu S. (2019). Chemometric assessment of spectroscopic techniques and antioxidant activity for *Hippophae rhamnoides* L. extracts obtained by different isolation methods. Anal. Lett..

[64] Tiwari D., Upmanyu N. (2021). Phytochemical analysis for bio-active potential of Semecarpus anacardium leaves. Plant Arch..

[65] Pandit S., Chang K.-W., Jeon J.-G. (2013). Effects of *Withania somnifera* on the growth and virulence properties of *Streptococcus mutans* and *Streptococcus sobrinus* at sub-MIC levels. Anaerobe.

[66] Zylber L.J., Jordan H.V. (1982). Development of a selective medium for detection and enumeration of *Actinomyces viscosus* and *Actinomyces naeslundii* in dental plaque. J. Clin. Microbiol..

[67] Saarela M., Hallamaa K., Mattila-Sandholm T., Mättö J. (2003). The effect of lactose derivatives lactulose, lactitol and lactobionic acid on the functional and technological properties of potentially probiotic Lactobacillus strains. Int. Dairy. J..

[68] Goderska K., Nowak J., Czarnecki Z. (2008). Comparision of growth of *Lactobacillus acidophilus* and *Bifidobacterium bifidum* species in media suplemented with selected saccharides including prebiotics. Acta Sci. Pol. Technol. Aliment..

[69] Holdeman L.V., Cato E.P., Burmeister J., Moore W. (1980). Descriptions of *Eubacterium timidum* sp. nov., *Eubacterium brachy* sp. nov., and *Eubacterium nodatum* sp. nov. isolated from human periodontitis. Int. J. Syst. Evol. Microbiol..

[70] Downes J., Wade W.G. (2009). *Propionibacterium acidifaciens* sp. nov., isolated from the human mouth. Int. J. Syst. Evol. Microbiol..

[71] Norris S.J., Paster B.J., Smibert R.M. (2015). Treponema. Bergey’s Manual of Systematics of Archaea and Bacteria.

[72] Cruickshank J., Neil-Dwyer G., Lane J. (1975). The effect of oral propranolol upon the ECG changes occurring in subarachnoid haemorrhage. Cardiovasc. Res..

[73] Saravia M.E., da Silva L.A.B., da Silva R.A.B., Cudmani N.M., Tineo S., Hillen N.E., Lucisano M.P., de Queiroz A.M., Emilson C.-G., Nelson-Filho P. (2020). Morphological identification of *Streptococcus mutans* and *Streptococcus sobrinus* in SB-20M culture medium has efficiency comparable to proteomic identification by the MALDI-TOF mass spectrometry technique. Arch. Oral. Biol..

[74] Peres M.A., Macpherson L.M., Weyant R.J., Daly B., Venturelli R., Mathur M.R., Listl S., Celeste R.K., Guarnizo-Herreño C.C., Kearns C. (2019). Oral diseases: A global public health challenge. Lancet.

[75] Reddy P., Krithikadatta J., Srinivasan V., Raghu S., Velumurugan N. (2020). Dental caries profile and associated risk factors among adolescent school children in an urban South-Indian city. Oral. Health Prev. Dent..

[76] Janakiram C., Antony B., Joseph J., Ramanarayanan V. (2018). Prevalence of Dental Caries in India among the WHO Index Age Groups: A Meta-Analysis. J. Clin. Diagn. Res..

[77] Siddiqui A.A., Alshammary F., Mulla M., Al-Zubaidi S.M., Afroze E., Amin J., Amin S., Shaikh S., Madfa A.A., Alam M.K. (2021). Prevalence of dental caries in Pakistan: A systematic review and meta-analysis. BMC Oral. Health.

[78] Giacaman R.A. (2018). Sugars and beyond. The role of sugars and the other nutrients and their potential impact on caries. Oral. Dis..

[79] Snyder M.L., Clarke M.K. (1950). Evaluation of the Colorimetric (Snyder) Test: I. Comparison of Positive Color Reactions with the Lactobacillus Counts of Respective Specimens of Saliva Routinely Submitted for Culture. J. Dent. Res..

[80] Qureshi F.H., Hamid S., Khan S.M., Qureshi A.H. (2018). Effect of tobacco use on tooth loss among patients visiting the out-patient dental department of a tertiary care hospital in Pakistan. JPMA.

[81] Chaitanya N.C., Boringi M., Madathanapalle R., Renee A., Sree S.V., Priyanka N., Sownetha T., Marella K. (2018). The prevalence of dental caries in smokers and smokeless tobacco users. Dent. Hypotheses.

[82] Ullah H., Ullah A., Khan M.W. (2020). Assessment of factors affecting and causing Hepatitis B in Balochistan-Pakistan. Pure Appl. Biol. PAB.

[83] Kaplan D.M. (2019). Food philosophy: An introduction.

[84] Kouidhi B., Al Qurashi Y.M.A., Chaieb K. (2015). Drug resistance of bacterial dental biofilm and the potential use of natural compounds as alternative for prevention and treatment. Microb. Pathog..

[85] Poveda Roda R., Bagán J.V., Sanchis Bielsa J.M., Carbonell Pastor E. (2007). Antibiotic use in dental practice: A review. Med. Oral Patol. Oral Y Cirugía Bucal Internet.

[86] Peedikayil F. (2011). Antibiotics: Use and misuse in pediatric dentistry. J. Indian. Soc. Pedod. Prev. Dent..

[87] Xu H., Tian J., Hao W., Zhang Q., Zhou Q., Shi W., Qin M., He X., Chen F. (2018). Oral microbiome shifts from caries-free to caries-affected status in 3-year-old Chinese children: A longitudinal study. Front. Microbiol..

[88] Yadav K., Prakash S. (2017). Dent caries: A microbiological approach. J. Clin. Infect. Dis. Pr..

[89] Wang Y.-L., Chang C.-C., Chi C.-W., Chang H.-H., Chiang Y.-C., Chuang Y.-C., Chang H.-H., Huang G.-F., Liao Y.-S., Lin C.-P. (2014). Erosive potential of soft drinks on human enamel: An in vitro study. J. Formos. Med. Assoc..

[90] Sohn W., Burt B.A., Sowers M.R. (2006). Carbonated soft drinks and dental caries in the primary dentition. J. Dent. Res..

[91] Soumya M., Nampoothiri K.M. (2021). An overview of functional genomics and relevance of glycosyltransferases in exopolysaccharide production by lactic acid bacteria. Int. J. Biol. Macromol..

[92] Hardy J.D., Webb W.R., Dalton M.L., Walker G.R. (1963). Lung homotransplantation in man: Report of the initial case. Jama.

[93] De Soet J., Van Loveren C., Lammens A., Pavičić M., Homburg C., Ten Cate J., De Graaff J. (1991). Differences in cariogenicity between fresh isolates of *Streptococcus sobrinus* and *Streptococcus mutans*. Caries Res..

[94] Elyassi M., Babaeekhou L., Ghane M. (2022). *Streptococcus mutans* and *Streptococcus sobrinus* contributions in dental caries in Iranian and Afghan children: A report from serotype distribution and novel STs. Arch. Oral. Biol..

[95] Okada M., Soda Y., Hayashi F., Doi T., Suzuki J., Miura K., Kozai K. (2005). Longitudinal study of dental caries incidence associated with *Streptococcus mutans* and *Streptococcus sobrinus* in pre-school children. J. Med. Microbiol..

[96] Gross E.L., Beall C.J., Kutsch S.R., Firestone N.D., Leys E.J., Griffen A.L. (2012). Beyond *Streptococcus mutans*: Dental caries onset linked to multiple species by 16S rRNA community analysis. PLoS ONE.

[97] Sissons C. (1997). Artificial dental plaque biofilm model systems. Adv. Dent. Res..

[98] Salako N., Kleinberg I. (1989). Incidence of selected ureolytic bacteria in human dental plaque from sites with differing salivary access. Arch. Oral. Biol..

[99] Agatonovic-Kustrin S., Gegechkori V., Mohammed E.U., Ku H., Morton D.W. (2022). Isolation of bioactive pentacyclic triterpenoid acids from olive tree leaves with flash chromatography. Appl. Sci..

[100] Qamar S., Torres Y.J., Parekh H.S., Falconer J.R. (2021). Extraction of medicinal cannabinoids through supercritical carbon dioxide technologies: A review. J. Chromatogr. B.

[101] Sultan I., Tareen M., Tareen A., Khan M. (2022). Phytochemical effectiveness of some ethanomedicinal plants of Balochistan, Pakistan against urogenital infections. Int. J. Agric. Technol..

[102] Rao J.K. (2021). Some Ethno-medicinal plants of Uttar Pradesh: A Review. Int. J. Biol. Innov..

[103] Sasidharan S., Chen Y., Saravanan D., Sundram K., Latha L.Y. (2011). Extraction, isolation and characterization of bioactive compounds from plants’ extracts. Afr. J. Tradit. Complement. Altern. Med..

[104] Patra J.K., Das G., Lee S., Kang S.-S., Shin H.-S. (2018). Selected commercial plants: A review of extraction and isolation of bioactive compounds and their pharmacological market value. Trends Food Sci. Technol..

[105] Kavya R., Shrungashree R., Suchitra S., Divakara R., Kekuda T. (2010). Comparative study on Antifungal activity and Proximate composition of Abrus pulchellus Wall and Abrus precatorius Linn. Res. J. Pharmacogn. Phytochem..

[106] Boggula N., Peddapalli H. (2017). Phytochemical analysis and evaluation of in vitro anti oxidant activity of Punica granatum leaves. Int. J. Pharmacogn. Phytochem. Res..

[107] Sharma K., Akansha C. (2018). Comparative studies of proximate, mineral and phytochemical compositions of pomegranate (Punica granatum) in peel, seed and whole fruit powder. Methods.

[108] Egamberdieva D., Jabborova D., Babich S., Xalmirzaeva S., Salakhiddinov K., Madazimov M. (2021). Antimicrobial activities of herbal plants from Uzbekistan against human pathogenic microbes. Environ. Sustain..

[109] Wolde T., Kuma H., Trueha K., Yabeker A. (2018). Anti-bacterial activity of garlic extract against human pathogenic bacteria. J. Pharmacovigil..

[110] Kshirsagar M.M., Dodamani A.S., Vishwakarma P., Mali G., Khobragade V.R., Deokar R.N. (2021). Comparative Assessment of Antibacterial Efficacy of Commercially Available Different Dental Gels: An in-vitro Study. Rev. Recent. Clin. Trials.

[111] Bhatwalkar S.B., Mondal R., Krishna S.B.N., Adam J.K., Govender P., Anupam R. (2021). Antibacterial properties of organosulfur compounds of garlic (*Allium sativum*). Front. Microbiol..

[112] Guidi L., Landi M. (2014). Aromatic plants: Use and nutraceutical properties. Nov. Plant Bioresour. Appl. Food Med. Cosmet..

[113] Fufa B.K. (2019). Anti-bacterial and anti-fungal properties of garlic extract (*Allium sativum*): A review. Microbiol. Res. J. Int..

[114] Chattopadhyay D., Dastidar S., Chakrabarty A. (1988). Antimicrobial properties of methdilazine and its synergism with antibiotics and some chemotherapeutic agents. Arzneim.-Forsch..

[115] Abdulrahman D.M., Daskum A.M., Abdulrahim K.M., Dadile A.M., Amma H. (2017). Antibacterial potency of garlic extract against certain skin pathogenic bacteria. Nov. Res. Microbiol. J..

[116] Kalkan S., Taş E., Erginkaya Z., Turhan E.Ü. (2017). Determination of antimicrobial effects of probiotic lactic acid bacteria and garlic extract against some foodborn pathogenic bacteria. Turk. J. Agric.-Food Sci. Technol..

[117] Guillamón E., Andreo-Martínez P., Mut-Salud N., Fonollá J., Baños A. (2021). Beneficial effects of organosulfur compounds from Allium cepa on gut health: A systematic review. Foods.

[118] Morshed M.S., Bhuyian A.M., Alam M.S., Belal M.T., Hossain S., Ali M.I., Zaman S.B. (2019). Outcomes of surgical management of fracture penis: Experience from a tertiary care hospital in bangladesh. PublicHealth Indones..

[119] Bouyahya A., Abrini J., Khay E.-O., Charfi S., Boujida N., EL-Harsal A., Talbaoui A., ET-Touys A., Bakri Y., Dakka N. (2016). In vitro antibacterial activity of organic extracts from north-west Moroccan medicinal plant *Myrtus communis* (L.). Biotechnol. J. Int..

[120] Kumbhani D., Goti D. (2020). Antimicrobial Activity of Fruits and Vegetables Peels on Human Enteric Pathogen: A Review. Int. J. Res. Publ. Rev..

[121] Aleem M., Khan M.I., Shakshaz F.A., Akbari N., Anwar D. (2020). Botany, phytochemistry and antimicrobial activity of ginger (*Zingiber officinale*): A review. Int. J. Herb. Med..

[122] Awano S., Ansai T., Takata Y., Soh I., Akifusa S., Hamasaki T., Yoshida A., Sonoki K., Fujisawa K., Takehara T. (2008). Oral health and mortality risk from pneumonia in the elderly. J. Dent. Res..

[123] Singh A.A., Naaz Z.T., Rakaseta E., Perera M., Singh V., Cheung W., Mani F., Nath S. (2023). Antimicrobial activity of selected plant extracts against common food borne pathogenic bacteria. Food Humanit..

[124] Hussain M.I., González L., Souto C., Reigosa M. (2011). Ecophysiological responses of three native herbs to phytotoxic potential of invasive *Acacia melanoxylon* R. Br. Agrofor. Syst..

[125] Variya B.C., Bakrania A.K., Patel S.S. (2016). *Emblica officinalis* (Amla): A review for its phytochemistry, ethnomedicinal uses and medicinal potentials with respect to molecular mechanisms. Pharmacol. Res..

[126] Thivaharan V., Ramesh V., Raja S. (2018). Green synthesis of silver nanoparticles for biomedical and environmental applications. Green. Met. Nanoparticles Synth. Charact. Their Appl..

[127] Ramanuj P.P., Granerød J., Davies N.W., Conti S., Brown D.W., Crowcroft N.S. (2014). Quality of life and associated socio-clinical factors after encephalitis in children and adults in England: A population-based, prospective cohort study. PLoS ONE.

[128] Pant D.R., Pant N.D., Yadav U.N., Khanal D.P. (2017). Phytochemical screening and study of antioxidant, antimicrobial, antidiabetic, anti-inflammatory and analgesic activities of extracts from stem wood of Pterocarpus marsupium Roxburgh. J. Intercult. Ethnopharmacol..

[129] Rahman H., Ansari M.I., Khangwal M., Solanki R., Mansoori S. (2021). Comparative evaluation of 6% cranberry, 10% green tea, 50% aloe vera and 10% sodium ascorbate on reversing the immediate bond strength of bleached enamel: In vitro study. J. Oral. Biol. Craniofacial Res..

[130] Kahramanoğlu İ., Chen C., Chen J., Wan C. (2019). Chemical constituents, antimicrobial activity, and food preservative characteristics of Aloe vera gel. Agronomy.

[131] Yousafzai A., Saleem S., Jahan N., Javed F., Khan M.W., Kanwal R. (2012). Extraction of Active Components of Aloe Vera to Treat Acne/pimple in Population of Quetta City. J. Appl. Emerg. Sci..

[132] Nabigol A., Asghari A. (2013). Antifungal activity of Aloe vera gel on quality of minimally processed pomegranate arils. Int. J. Agron. Plant Prod..

[133] Firdous N., Khan M.R., Butt M.S., Shahid M. (2020). Application of aloevera gel based edible coating to maintain postharvest quality of tomatoes. Pak. J. Agric. Sci..

[134] Igarashi T., Yamamoto A., Goto N. (2000). PCR for detection and identification of *Streptococcus sobrinus*. J. Med. Microbiol..

[135] Morou-Bermudez E., Burne R.A. (1999). Genetic and physiologic characterization of urease of *Actinomyces naeslundii*. Infect. Immun..

[136] Farid W., Masud T., Sohail A., Naqvi S., Qazalbash M.A. (2016). Molecular characterization and 16S rRNA sequence analysis of probiotic lactobacillus acidophilus isolated from indigenous Dahi (Yoghurt). Int. J. Biosci..

[137] Sato T., Sato M., Matsuyama J., Kalfas S., Sundqvist G., Hoshino E. (1998). Restriction fragment-length polymorphism analysis of 16S rDNA from oral asaccharolytic Eubacterium species amplified by polymerase chain reaction. Oral Microbiol. Immunol..

[138] Casal C.A.D., Silva M.O.d., Costa I.B., Araújo E.d.C., Corvelo T.C.d.O. (2011). Molecular detection of Treponema pallidum sp. pallidum in blood samples of VDRL-seroreactive women with lethal pregnancy outcomes: A retrospective observational study in northern Brazil. Rev. Soc. Bras. Med. Trop..

