# Supplementary material for: Phenotypic Analysis, Molecular Characterization, and Antibiogram of Caries-Causing Bacteria Isolated from Dental Patients
Source: Microorganisms. 2023 Jul 31;11(8):1952. doi: 10.3390/microorganisms11081952 (PMC10457851; doi:10.3390/microorganisms11081952)
Supplement: Supplementary file 1 [file microorganisms-11-01952-s001.zip › microorganisms-2500180-supplementary.pdf]

Table S1. Primers used in PCR amplification of conserved region in 16S rDNA gene for bacterial strains.

Molecular characterization	Size	Primers	Tm (° C)	PCR Reaction mixture	Reference
S. sobrinus	1610 bp	F-5'-TGCTATCTTTCCCTAGCATG-3'<br>R-5′-GGTATTCGGTTTGACTGC-3′	55	10 mM Tris-HCl, 1.5 mM $MgCl_2$, 50 mM KCl, 1mg gelatin, 200 μM dNTPs, 50 pmol primers, 10 μl of template solution, 2.5 U *Taq* DNA polymerase	[1]
S.mutans	1272 bp	F-5'-TATGCTGCTATTGGAGGTTC-3'<br>R-5'-AAGGTTGAGCAATTGAATCG-3'	55	10 mM Tris-HCl, 1.5 mM $MgCl_2$, 50 mM KCl, 1mg gelatin, 200 μM dNTPs, 50 pmol primers, 10 μl of template solution, 2.5 U *Taq* DNA polymerase	[1]
A. naeslundii	600bp	F-5′-AARATHCAYGARGAYTGGGG-3′<br>R-5′-GCNGGRTTDATNGTRTAYTT-3′	55	1.5 mM $MgCl_2$, 75 μM dNTPs, 2.5 U *Taq* DNA polymerase, 100 pmol primers, 0.5 μg of chromosomal DNA	[2]
L. acidophilus	1500 bp	F-5′-GGCTACCTTGTTACGA-3'<br>R-5′-GAGTTTGATCCTGGCTCAG-3'	56	2 μl DNA template, 1 μl reaction buffer, 0.6 μl $MgCl_2$, 0.5 μl primers, 0.4 μl *Taq* DNA polymerase, 0.2 μl dNTPs, 4.8 μl Nano pure water	[3]
E. nodatum	492 bp	F-5'-AGA GTT TGA TCC TGG CTC AG-3'<br>R-5'-GCC TTA AAC CCT RCG CTT-3'	52	4 μl DNA template, 2 μl reaction buffer, 0.6 μl $MgCl_2$, 0.5 μl primers, 0.4 μl *Taq* DNA polymerase, 0.2 μl dNTPs, 12.3 μl Nano pure water	[4]
P. acidifaciens	950 bp	F-5'-GCACCTTCATYATCAACGGCAC-3'<br>R-5'-CACATVCGRCCGTAGTG-3'	56	5 μl DNA template, 2 μl reaction buffer, 0.6 μl $MgCl_2$, 0.5 μl primers, 0.4 μl *Taq* DNA polymerase, 0.2 μl dNTPs, 16.3 μl Nano pure water	[5]

T. pallidum	209 bp	F-5′-AGACGGCTGCACATCTTCTCCA-3′ R-5′-AGCAGACGTTACATCGAGCGGA-3′	60	1X PCR buffer, 0.18 mM $MgCl_2$, 5 µl DNA template, 0.02 mM dNTPs, U *Taq* DNA platinum polymerase, Up to the final volume added sterile water	[6]

Table S2. ANOVA for antibacterial activity of herbal extracts for *S. sobrinus.*

**Two-way RM ANOVA**	**Matching: Both factors**				
Assume sphericity?	No				
Alpha	0.05				
Source of Variation	% of total variation	*p* value	*p* value summary	Significant?	Geisser-Greenhouse's epsilon
Strength of extract	13.48	<0.0001	****	Yes	0.1124
Plant	17.35	<0.0001	****	Yes	0.2212
Strength of extract x Plant	69.05	<0.0001	****	Yes	0.02574
Type of extracts x Strength of extract	0.004441				
Type of extracts x Plant	0.002476				
Type of extracts	0.08958				
ANOVA table	SS	DF	MS	F (DFn, DFd)	*p* value
Strength of extract	280.3	11	25.48	F (1.236, 2.472) = 6069	*p* <0.0001
Plants	360.8	5	72.16	F (1.106, 2.212) = 14017	*p* <0.0001
Strength of extract x Plant	1436	55	26.11	F (1.416, 2.831) = 5746	*p* <0.0001
Type of extracts x Strength of extract	0.09236	22	0.004198		
Type of extracts x Plant	0.05148	10	0.005148		
Type of extracts	1.863	2	0.9314		
Residual	0.4998	110	0.004544		
Data summary					
Plant	6				
Strength of extract	12				
Type of extracts	3				
Number of missing values	0				

Table S3. ANOVA for antibacterial activity of herbal extracts for *S. mutans*.

**Two-way RM ANOVA**	**Matching: Both factors**				
Assume sphericity?	No				
Alpha	0.05				
Source of Variation	% of total variation	*p* value	*p* value summary	Significant?	Geisser-Greenhouse's epsilon
Strength of extract	13.48	<0.0001	****	Yes	0.1124
Plant	17.35	<0.0001	****	Yes	0.2212
Strength of extract x Plant	69.05	<0.0001	****	Yes	0.02574
Type of extracts x Strength of extract	0.004441				
Type of extracts x Plant	0.002476				
Type of extracts	0.08958				
ANOVA table	SS	DF	MS	F (DFn, DFd)	*p* value
Strength of extract	280.3	11	25.48	F (1.236, 2.472) = 6069	*p* <0.0001
Plants	360.8	5	72.16	F (1.106, 2.212) = 14017	*p* <0.0001
Strength of extract x Plant	1436	55	26.11	F (1.416, 2.831) = 5746	*p* <0.0001
Type of extracts x Strength of extract	0.09236	22	0.004198		
Type of extracts x Plant	0.05148	10	0.005148		
Type of extracts	1.863	2	0.9314		
Residual	0.4998	110	0.004544		
Data summary					
Plant	6				
Strength of extract	12				
Type of extracts	3				
Number of missing values	0				

Table S4. ANOVA for antibacterial activity of herbal extracts for *A. naeslundii.*

**Two-way RM ANOVA**	**Matching: Both factors**				
Assume sphericity?	No				
Alpha	0.05				
Source of Variation	% of total variation	*p* value	*p* value summary	Significant?	Geisser-Greenhouse's epsilon
Strength of extract	10.92	<0.0001	****	Yes	0.131
Plant	56.6	<0.0001	****	Yes	0.2324
Strength of extract x Plant	32.38	<0.0001	****	Yes	0.03286
Type of extracts x Strength of extract	0.003634				
Type of extracts x Plant	0.003737				
Type of extracts	0.08416				
ANOVA table	SS	DF	MS	F (DFn, DFd)	*p* value
Strength of extract	182.9	11	16.63	F (1.440, 2.881) = 6008	*p* <0.0001
Plants	948.4	5	189.7	F (1.162, 2.324) = 30290	*p* <0.0001
Strength of extract x Plant	542.7	55	9.867	F (1.807, 3.614) = 4837	*p* <0.0001
Type of extracts x Strength of extract	0.0609	22	0.002768		
Type of extracts x Plant	0.06262	10	0.006262		
Type of extracts	1.41	2	0.7052		
Residual	0.2244	110	0.00204		
Data summary					
Plant	6				
Strength of extract	12				
Type of extracts	3				
Number of missing values	0				

Table S5. ANOVA for antibacterial activity of herbal extracts for *L. acidophilus.*

**Two-way RM ANOVA**	**Matching: Both factors**				
Assume sphericity?	No				
Alpha	0.05				
Source of Variation	% of total variation	*p* value	*p* value summary	Significant?	Geisser-Greenhouse's epsilon
Strength of extract	10.4	<0.0001	****	Yes	0.1075
Plant	56.45	<0.0001	****	Yes	0.2222
Strength of extract x Plant	33.03	<0.0001	****	Yes	0.02633
Type of extracts x Strength of extract	0.002761				
Type of extracts x Plant	0.00514				
Type of extracts	0.09129				
ANOVA table	SS	DF	MS	F (DFn, DFd)	*p* value
Strength of extract	185.5	11	16.87	F (1.182, 2.365) = 7529	*p* <0.0001
Plants	1008	5	201.5	F (1.111, 2.222) = 21966	*p* <0.0001
Strength of extract x Plant	589.6	55	10.72	F (1.448, 2.896) = 3820	*p* <0.0001
Type of extracts x Strength of extract	0.04928	22	0.00224		
Type of extracts x Plant	0.09174	10	0.009174		
Type of extracts	1.629	2	0.8146		
Residual	0.3087	110	0.002806		
Data summary					
Plant	6				
Strength of extract	12				
Type of extracts	3				
Number of missing values	0				

Table S6. ANOVA for antibacterial activity of herbal extracts for *E. nodatum***.**

Two-way RM ANOVA	Matching: Both factors				
Assume sphericity?	No				
Alpha	0.05				
Source of Variation	% of total variation	*p* value	*p* value summary	Significant?	Geisser-Greenhouse's epsilon
Strength of extract	10.38	<0.0001	****	Yes	0.112
Plant	57.57	<0.0001	****	Yes	0.2151
Strength of extract x Plant	31.92	<0.0001	****	Yes	0.02427
Type of extracts x Strength of extract	0.005656				
Type of extracts x Plant	0.006558				
Type of extracts	0.1041				
ANOVA table	SS	DF	MS	F (DFn, DFd)	*p* value
Strength of extract	171.6	11	15.6	F (1.233, 2.465) = 3669	*p* <0.0001
Plants	952.1	5	190.4	F (1.075, 2.151) = 17555	*p* <0.0001
Strength of extract x Plant	527.8	55	9.597	F (1.335, 2.669) = 2837	*p* <0.0001
Type of extracts x Strength of extract	0.09354	22	0.004252		
Type of extracts x Plant	0.1085	10	0.01085		
Type of extracts	1.721	2	0.8607		
Residual	0.3721	110	0.003383		
Data summary					
Plant	6				
Strength of extract	12				
Type of extracts	3				
Number of missing values	0				

Table S7. ANOVA for antibacterial activity of herbal extracts for *P. acidifaciens*.

**Two-way RM ANOVA**	**Matching: Both factors**				
Assume sphericity?	No				
Alpha	0.05				
Source of Variation	% of total variation	*p* value	*p* value summary	Significant?	Geisser-Greenhouse's epsilon
Strength of extract	12.17	<0.0001	****	Yes	0.131
Plant	56.78	<0.0001	****	Yes	0.2209
Strength of extract x Plant	30.92	<0.0001	****	Yes	0.02727
Type of extracts x Strength of extract	0.00405				
Type of extracts x Plant	0.003213				
Type of extracts	0.09746				
ANOVA table	SS	DF	MS	F (DFn, DFd)	*p* value
Strength of extract	211.6	11	19.24	F (1.441, 2.881) = 6012	*p* <0.0001
Plants	987.1	5	197.4	F (1.105, 2.209) = 35341	*p* <0.0001
Strength of extract x Plant	537.6	55	9.775	F (1.500, 3.000) = 3562	*p* <0.0001
Type of extracts x Strength of extract	0.0704	22	0.0032		
Type of extracts x Plant	0.05586	10	0.005586		
Type of extracts	1.694	2	0.8471		
Residual	0.3019	110	0.002744		
Data summary					
Plant	6				
Strength of extract	12				
Type of extracts	3				
Number of missing values	0				

Table S8. ANOVA for antibacterial activity of herbal extracts for *T. pallidum.*

**Two-way RM ANOVA**	**Matching: Both factors**				
Assume sphericity?	No				
Alpha	0.05				
Source of Variation	% of total variation	*p* value	*p* value summary	Significant?	Geisser-Greenhouse's epsilon
Strength of extract	12.44	<0.0001	****	Yes	0.1411
Plant	56.87	<0.0001	****	Yes	0.2124
Strength of extract x Plant	30.56	<0.0001	****	Yes	0.02792
Type of extracts x Strength of extract	0.003487				
Type of extracts x Plant	0.005061				
Type of extracts	0.09802				
ANOVA table	SS	DF	MS	F (DFn, DFd)	*p* value
Strength of extract	218.4	11	19.86	F (1.553, 3.105) = 7138	*p* <0.0001
Plants	998.1	5	199.6	F (1.062, 2.124) = 22472	*p* <0.0001
Strength of extract x Plant	536.4	55	9.754	F (1.536, 3.072) = 3038	*p* <0.0001
Type of extracts x Strength of extract	0.06121	22	0.002782		
Type of extracts x Plant	0.08883	10	0.008883		
Type of extracts	1.721	2	0.8603		
Residual	0.3532	110	0.00321		
Data summary					
Plant	6				
Strength of extract	12				
Type of extracts	3				
Number of missing values	0				

References

1. Igarashi, T.; Yamamoto, A.; Goto, N. PCR for detection and identification of Streptococcus sobrinus. *Journal of medical microbiology* **2000**, *49*, 1069-1074.
2. Morou-Bermudez, E.; Burne, R.A. Genetic and physiologic characterization of urease of Actinomyces naeslundii. *Infection and immunity* **1999**, *67*, 504-512.
3. Farid, W.; Masud, T.; Sohail, A.; Naqvi, S.; Qazalbash, M.A. Molecular characterization and 16S rRNA sequence analysis of probiotic lactobacillus acidophilus isolated from indigenous Dahi (Yoghurt). *International Journal of Bioscience* **2016**, *9*, 19-27.
4. Sato, T.; Sato, M.; Matsuyama, J.; Kalfas, S.; Sundqvist, G.; Hoshino, E. Restriction fragment - length polymorphism analysis of 16S rDNA from oral asaccharolytic Eubacterium species amplified by polymerase chain reaction. *Oral microbiology and immunology* **1998**, *13*, 23-29.
5. Downes, J.; Wade, W.G. Propionibacterium acidifaciens sp. nov., isolated from the human mouth. *International journal of systematic and evolutionary microbiology* **2009**, *59*, 2778-2781.
6. Casal, C.A.D.; Silva, M.O.d.; Costa, I.B.; Araújo, E.d.C.; Corvelo, T.C.d.O. Molecular detection of Treponema pallidum sp. pallidum in blood samples of VDRL-seroreactive women with lethal pregnancy outcomes: a retrospective observational study in northern Brazil. *Revista da Sociedade Brasileira de Medicina Tropical* **2011**, *44*, 451-456.
